# Structures and Reactivities of Cocrystals Involving Diboronic Acids and Bipyridines: In Situ Linker Reaction and 1D‐to‐2D Dimensionality Change via Crystal‐to‐Crystal Photodimerization

**DOI:** 10.1002/chem.202104604

**Published:** 2022-03-25

**Authors:** María Guadalupe Vasquez‐Ríos, Gonzalo Campillo‐Alvarado, Dale C. Swenson, Herbert Höpfl, Leonard R. MacGillivray

**Affiliations:** ^1^ Department of Chemistry University of Iowa Iowa City, 1A 52242-1294 USA; ^2^ Department of Chemical and Biomolecular Engineering University of Illinois Urbana-Champaign 600 S. Mathews Avenue Urbana IL 61801 USA; ^3^ Centro de Investigaciones Químicas Instituto de Investigación en Ciencias Básicas y Aplicadas (IICBA) Universidad Autónoma del Estado de Morelos Av. Universidad 1001 62209 Cuernavaca Morelos (México

**Keywords:** [2+2] photodimerization, bipyridines, cocrystals, diboronic acids, in situ linker transformation

## Abstract

Cocrystallizations of diboronic acids [1,3‐benzenediboronic acid (1,3‐bdba), 1,4‐benzenediboronic acid (1,4‐bdba) and 4,4’‐biphenyldiboronic acid (4,4’‐bphdba)] and bipyridines [1,2‐bis(4‐pyridyl)ethylene (bpe) and 1,2‐bis(4‐pyridyl)ethane (bpeta)] generated the hydrogen‐bonded 1 : 2 cocrystals [(1,4‐bdba)(bpe)_2_] (1), [(1,4‐bdba)(bpeta)_2_] (2), [(1,3‐bdba)(bpe)_2_(H_2_O)_2_] (3) and [(1,3‐bdba)(bpeta)_2_(H_2_O)] (4), wherein 1,3‐bdba involved hydrated assemblies. The linear extended 4,4’‐bphdba exhibited the formation of 1 : 1 cocrystals [(4,4'‐bphdba)(bpe)] (5) and [(4,4'‐bphdba‐me)(bpeta)] (6). For 6, a hemiester was generated by an in‐situ linker transformation. Single‐crystal X‐ray diffraction revealed all structures to be sustained by B(O)−H⋅⋅⋅N, B(O)−H⋅⋅⋅O, O_w_−H⋅⋅⋅O, O_w_−H⋅⋅⋅N, C−H⋅⋅⋅O, C−H⋅⋅⋅N, π⋅⋅⋅π, and C−H⋅⋅⋅π interactions. The cocrystals comprise 1D, 2D, and 3D hydrogen‐bonded frameworks with components that display reactivities upon cocrystal formation and within the solids. In 1 and 3, the C=C bonds of the bpe molecules undergo a [2+2] photodimerization. UV radiation of each compound resulted in quantitative conversion of bpe into cyclobutane tpcb. The reactivity involving 1 occurred via 1D‐to‐2D single‐crystal‐to‐single‐crystal (SCSC) transformation. Our work supports the feasibility of the diboronic acids as formidable structural and reactivity building blocks for cocrystal construction.

## Graphical abstract

**Chemical reactivity** results in an in‐situ linker modification and a switch of dimensionality with light involving cocrystals of diboronic acids and bipyridines.

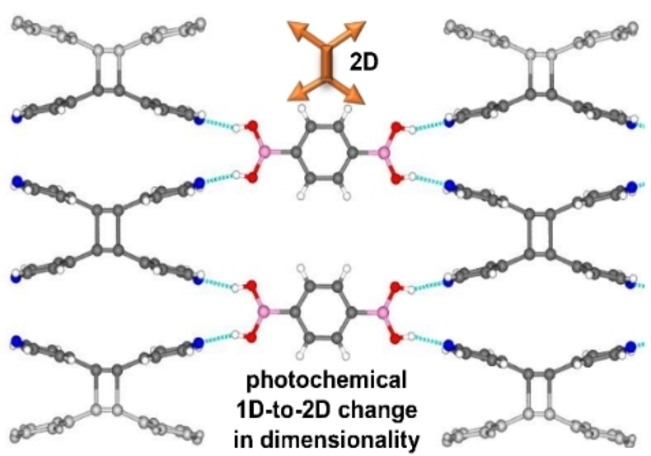

## Introduction

Self‐assembly is a ubiquitous process in Nature that enables systems to adapt to environmental changes.^[1]^ At the molecular level, adaptation originates from weak, non‐covalent interactions and is subject to supramolecular synthons encoded in molecular structures and the process of molecular recognition.[^2^, ^3^, ^4^, ^5^, ^6^, ^7^] In this context, boronic acids are highly versatile building blocks to develop functional supramolecular materials (for example, saccharide sensors,^[8]^ pharmaceutics,[^9^, ^10^, ^11^, ^12^] porous,^[13]^ and photoactive solids[^14^, ^15^]). The acid moiety, for example, has a capacity to accommodate conformational changes in the B(OH)_2_ group (that is, *syn‐syn*, *anti‐syn*, *anti‐anti*) upon hydrogen‐bond mediated self‐assembly.[^16^, ^17^, ^18^] The acid group also exhibits a preference to function as a DD (where: D=hydrogen‐bond donor) module for molecular recognition with appropriate AA moieties (where: A=hydrogen‐bond acceptor).[^19^, ^20^] Collectively, the conformational flexibility and hydrogen‐bonding capabilities allow for hydrogen‐bonded substrates to be assembled and organized in close proximity in the surrounding environment of the acid.^[21]^ While conformational landscapes of boronic acids in the solid state are now becoming established, information concerning the self‐assembly of diboronic acids as related to hydrogen‐bond mediated self‐assembly has yet to be extensively addressed.^[20]^

In this context, reports by Bonifazi et al. exploit self‐adapting capacities of diboronic acids to achieve supramolecular polymers^[19]^ and discrete assemblies as DD units through H‐bonding with AA N‐acceptors.^[20]^ The studies demonstrate diboronic acid recognition in the presence of complementary acceptors. The work follows earlier studies by Pedireddi and Höpfl involving cocrystals of monoboronic and diboronic acids with 4,4’‐bipyridine (4,4’‐bpy) and 1,2‐bis(4‐pyridyl)ethylene (bpe).[^22^, ^23^]

**Scheme 1:**
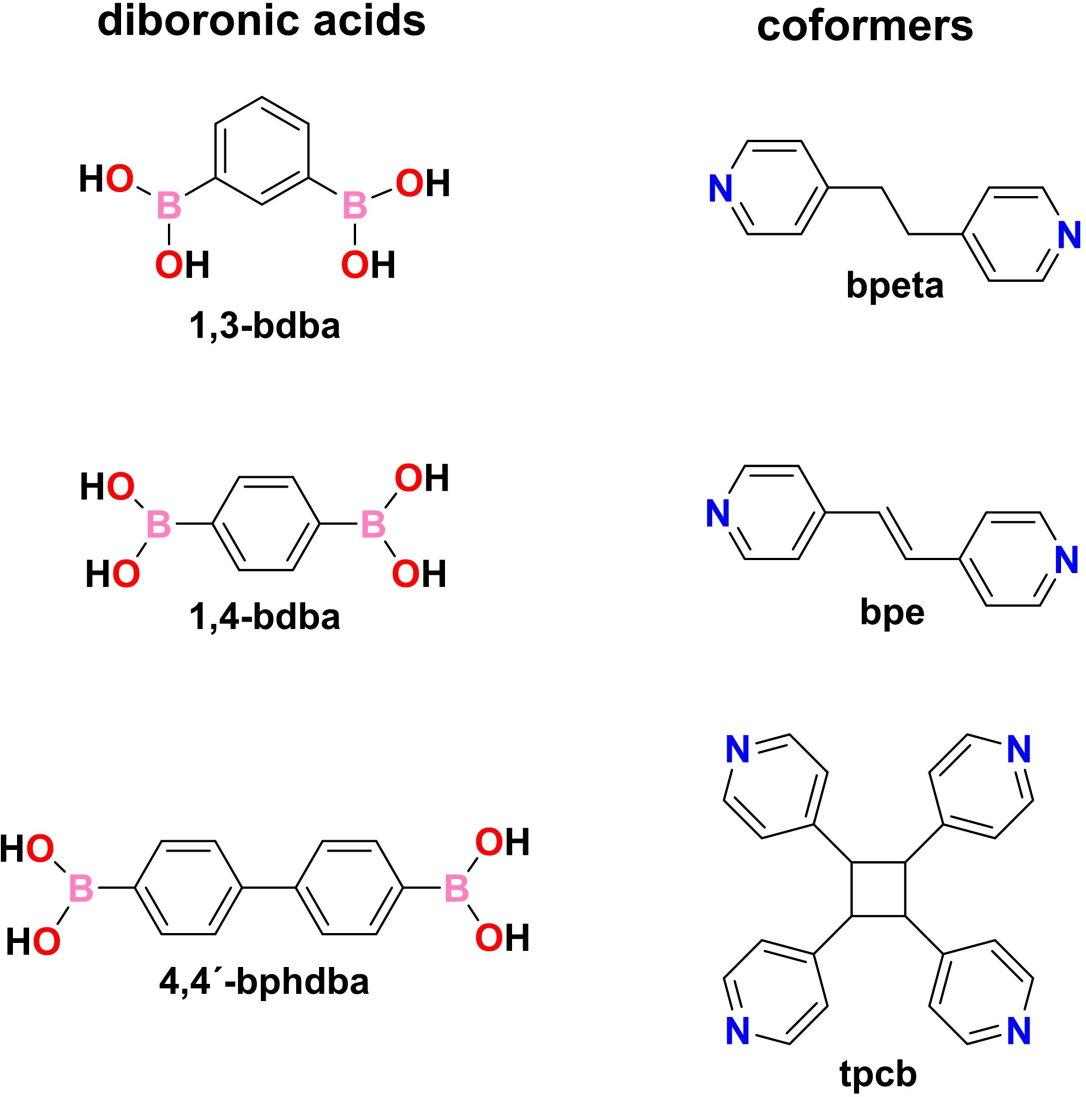
Diboronic acids and bipyridine coformers studied.

Given our recent studies involving cocrystals of monoboronic acids with N‐acceptor bipyridines,[^24^, ^25^] we sought to explore the capacity of changes to the structures of diboronic acids [that is, 1,3‐benzenediboronic acid (1,3‐bdba), 1,4‐benzenediboronic acid (1,4‐bdba) and 4,4’‐biphenyldiboronic acid (4,4’‐bphdba)] and bipyridine coformers [that is, alkane vs. alkene groups of bpe and 1,2‐bis(4‐pyridyl)ethane (bpeta)] to result in conformational and assembly variations of the B(OH)_2_ groups. The variations are expected to trigger adaptability of boronic acids in the self‐assembly process (Scheme 1).^[22]^

Our work involving cocrystals of boronic acids is also motivated by ongoing interests to direct photodimerizations in organic solids and, more generally, to learn how principles of supramolecular chemistry can affect and control chemical reactivity in the crystalline state. We identified monoboronic acids to function as hydrogen‐bond‐donor DD templates that direct intermolecular [2+2] photodimerizations of bpe to generate tpcb. After photoreaction, the resulting cyclobutane tpcb functions as an AA acceptor as evidenced by single‐crystal reactivity properties.^[14]^ Monoboronic acids can now be regarded as tools to control photocycloaddition in the solid state, although efforts are necessary to probe the scope of the self‐assembly process and templating behavior. We have also demonstrated the ability of related boronic esters to serve as templates of monopyridines in the form of stilbazoles.^[26]^

**Scheme 2:**
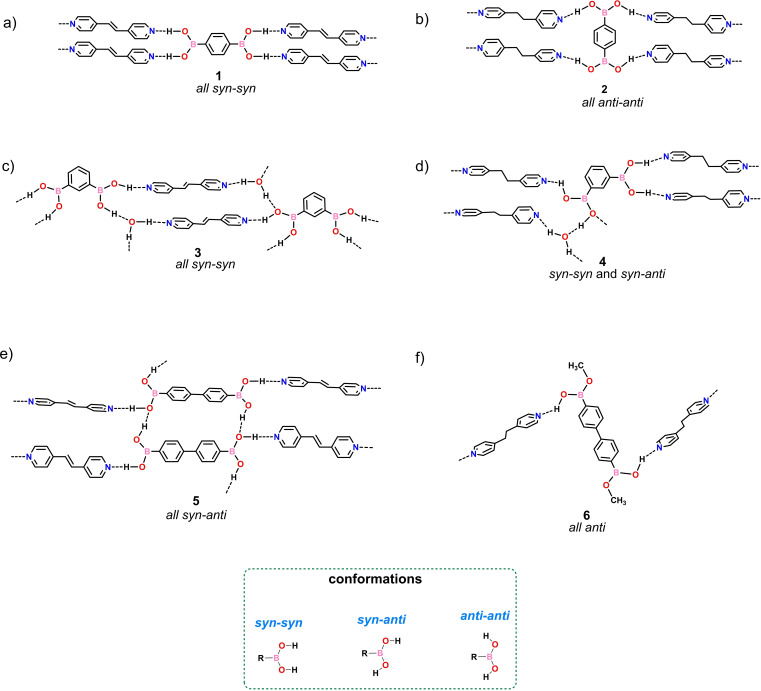
Hydrogen‐bonded architectures involving diboronic acids (1,3‐bdba, 1,4‐bdba and 4,4'‐bphdba) and bipyridines (bpeta and bpe).

Here, we report the self‐assembly of diboronic acids and the extended bipyridines bpe and bpeta showing that subtle changes to constituent components lead to broad diversification of cocrystal composition, supramolecular architectures, crystalline motifs, and reactivities of components in both the solid state and liquid phase. We show linear 1,4‐bdba to display *all syn‐syn* and *all anti‐anti* conformations in the 1 : 2 cocrystals [(1,4‐bdba)(bpe)_2_] (1) and [(1,4‐bdba)(bpeta)_2_] (2), respectively, generating 1D chains (Scheme 2a and b). For angular 1,3‐bdba, the cocrystals exhibit hydrated 1 : 2 : 2 and 1 : 2 : 1 supramolecular assemblies^[22]^ where the components of [(1,3‐bdba)(bpe)_2_(H_2_O)_2_] (3) interact through *all syn‐syn* conformations whereas in [(1,3‐bdba)(bpeta)_2_(H_2_O)] (4) both *syn‐syn* and *syn‐anti* conformations are present (Scheme 2c and d). For linear extended 4,4’‐bphdba, an *all syn‐anti* conformation in the 1 : 1 cocrystal [(4,4'‐bphdba)(bpe)] (5) supports a 1D polymer. For the saturated bipyridine, the extended diacid underwent an in‐situ esterification with methanol upon cocrystallization to give [(4,4'‐bphdba‐me)(bpeta)] (6) as a 1D polymer with the diester in an *all anti* conformation (Scheme 2e and f). UV irradiation of both 1 and 3,^[27]^ generates the cyclobutane derivative tpcb stereoselectively and in quantitative yield. For 1, the reaction in the solid state occurs in a single‐crystal‐to‐single‐crystal (SCSC) transformation wherein the dimensionality of the polymer undergoes a 1D‐to‐2D change to generate [(1,4‐bdba)(tpcb)] (1R). In the photoreacted solid, the photoproduct tpcb interacts with four different diboronic acids.

## Results and Discussion

Our original aim was to compare structural hydrogen‐bonding motifs containing diboronic acids (1,3‐bdba, 1,4‐bdba and 4,4'‐bphdba) and bipyridines (bpeta and bpe). The ditopic hydrogen‐bond‐acceptors were selected based on structural effects that arise owing to the different hybridizations of the central carbon‐carbon linkage (sp^3^ vs. sp^2^). To synthesize the cocrystals, the corresponding diboronic acid was placed in a vial with methanol and acetone as solvents. Each bipyridine was then added in a 1 : 2 stoichiometry and the solution stirred for approximately 10 min at room temperature. Crystals suitable for single‐crystal X‐ray diffraction (SCXRD) analysis were achieved by slow solvent evaporation (Figures S1–S7, see Supporting Information). Relevant crystallographic data are summarized in Tables S1–S7. Phase purities were established by comparisons of calculated and experimental powder X‐ray diffraction analysis (PXRD) patterns (Figures S8–S13, see Supporting Information). The solid products were also characterized by ^1^H NMR spectroscopy in solution (Figures S15–S20, see Supporting Information).

## Single‐crystal X‐ray diffraction analyses

### Assemblies of 1,4‐bdba and single‐crystal reactivity with bpe

**Figure 1:**
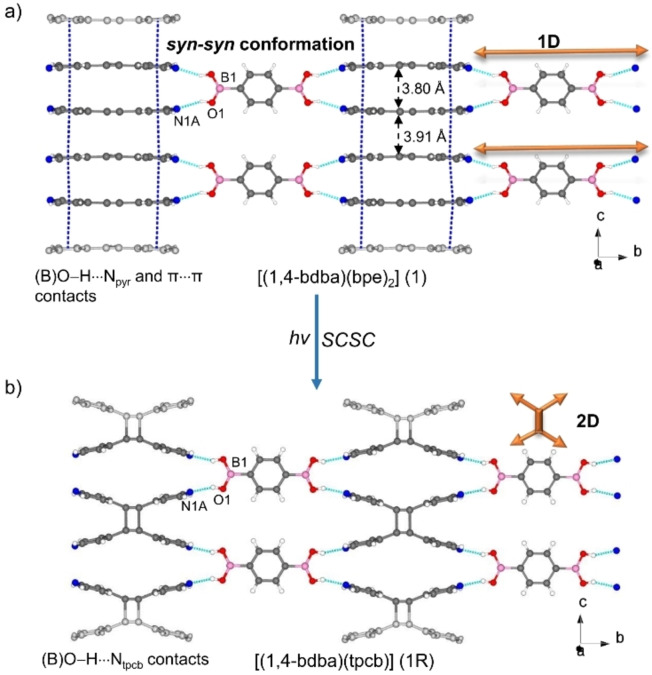
X‐ray structure of 1R from SCSC reaction of 1: a) 1D hydrogen‐bonded polymer of 1, b) 2D hydrogen‐bonded framework of 1R (disorder omitted for clarity).

Cocrystallizations of the linear diacid 1,4‐bdba with bpe and bpeta afforded [(1,4‐bdba)(bpe)_2_] (1) and [(1,4‐bdba)(bpeta)_2_] (2), respectively. The components of 1 crystallize in the monoclinic space group *C2/m*, with the asymmetric unit consisting of a quarter molecule of 1,4‐bdba and a half molecule of bpe (Figure S1, see Supporting Information) to give a 1D supramolecular arrangement sustained by B(O)−H⋅⋅⋅N_pyr_ hydrogen bonds [O⋅⋅⋅N, 2.735(14) Å/2.860(12) Å; ∠O−H⋅⋅⋅N 155°/152°]. The diacid adopts the *all syn‐syn* conformation such that the bpe molecules assemble as face‐to‐face π‐stacked dimers within the 1D structure [centroid‐centroid 3.59 Å and N⋅⋅⋅N 3.527(4) Å]. The conformation is akin to a DD hydrogen‐bond donor module.^[19]^ The B(OH)_2_ groups lie coplanar (0.08°) with the pendant aromatic ring while the bpe and 1,4‐bdba rings are twisted toward orthogonal (64°). The pyridyl rings of bpe are approximately coplanar (twist angle ∼5.73°). The stacking of bpe generates infinite face‐to‐face π stacks with the combined hydrogen bonding and stacking producing a 2D arrangement within the *bc*‐plane (Figure 1a). The C=C bonds of bpe lie disordered over two sites (occupancies: 0.45/0.55 at temperature 298 K). The stacked alkenes are parallel with the C=C bonds separated by 3.80 Å and 3.91 Å, which is a geometry suitable for an intermolecular [2+2] photodimerization.^[27]^

When single crystals of 1 were subjected to UV irradiation for 3 h (450 W, medium‐pressure Hg‐lamp), bpe reacted to form [(1,4‐bdba)(tpcb)] (1R) in quantitative yield as confirmed by ^1^H NMR spectroscopy (Figure S21; see Supporting Information). The formation of tpcb was evidenced by the disappearance of the olefinic signals (7.54 ppm) and appearance of cyclobutane signals (4.66 ppm). Visual examination suggested the single crystals to undergo a SCSC photoreaction.

A SCXRD analysis of the photoreacted solid 1R revealed components of the cocrystal to undergo a SCSC transformation.

The components occupy the same monoclinic space group *C2/m*, with the asymmetric unit consisting of a quarter molecule of 1,4‐bdba and a quarter molecule of tpcb. The cyclobutane lies disordered on a two‐fold rotation axis (occupancies: 0.45/0.55) (Figure S2, see Supporting Information). The cell volume of 1R increased on the order of 1.6 % versus the unreacted solid.

The photodimerization resulted in a structural modification of the DD recognition properties of the −B(OH)_2_ group. Specifically, the photoreaction involved the larger of the stacked C=C bond separations (that is, 3.91 compared to 3.80) (Figure 1).^[28]^ As a result, each −B(OH)_2_ group in the *all syn‐syn* conformation of the diacid interacts with two different cyclobutane molecules, interacting with tpcb via B(O)−H⋅⋅⋅N_tpcb_ hydrogen bonds [O⋅⋅⋅N 2.742(15) Å/2.778(15) Å; ∠O−H⋅⋅⋅N 160°/156°; N⋅⋅⋅N 3.308(3) Å]. Owing to the photoreaction, the components participate in a 2D hydrogen‐bonded polymer in the *bc*‐plane. The SCSC transformation, thus, involved a 1D‐to‐2D change in dimensionality of the hydrogen bonds.^[29]^ To our knowledge, 1R is the first example of a hydrogen‐bonded architecture based on a diboronic acid that undergoes a change in dimensionality. There is, thus, a need to consider changes in dimensionally that can occur when engineering topochemically reactive alkenes in solids based on the −B(OH)_2_ group. The observations also attest to the conformational flexibility of the −B(OH)_2_ group.

**Figure 2:**
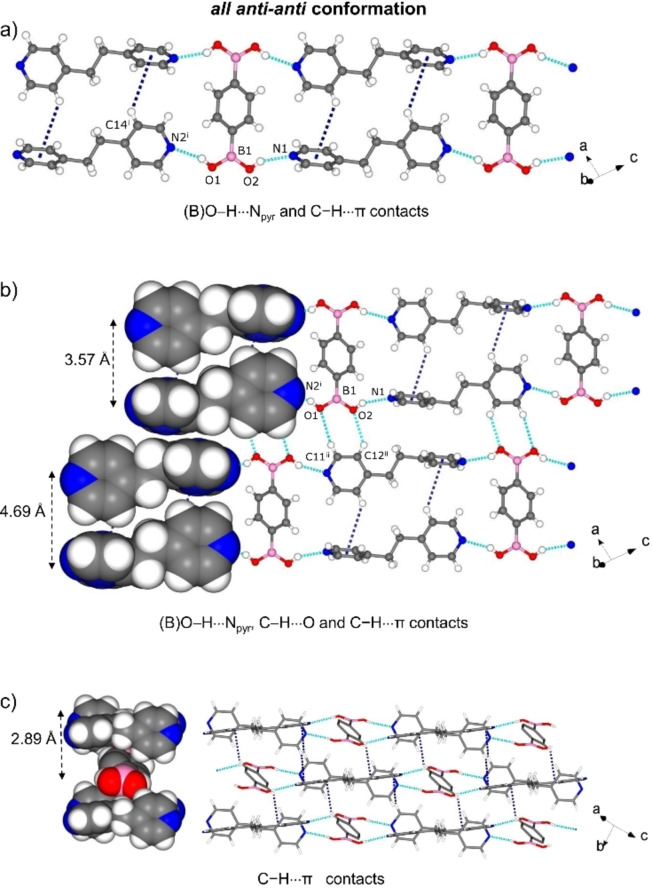
X‐ray structure of 2: a) 1D strands based on (B)O−H⋅⋅⋅N_pyr_ and C−H⋅⋅⋅π interactions, b) 2D hydrogen bonded framework in *ac*‐plane, and c) 3D network through additional C−H⋅⋅⋅π contacts. Symmetry operators: i) 1+*x*, 1+*y*, −1+*z*; ii) −*x*, 1+*y*, 1−*z*.

The components of 2 crystallize in the triclinic space group *P‐1* with one molecule of bpeta and half of 1,4‐bdba in the asymmetric unit (Figure S3, see Supporting Information). In contrast to 1, the diboronic acid adopts an *all anti‐anti* conformation and the long molecular axes of 1,4‐bdba and the coformer are oriented perpendicular to each other, instead of parallel. Similar to 1, the components form a 1D supramolecular arrangement in the *ac*‐plane sustained by B(O)−H⋅⋅⋅N_pyr_ [O⋅⋅⋅N, 2.821(2) Å/2.806(2) Å; ∠O−H⋅⋅⋅N, 158°/154°] hydrogen bonds (Figure 2a), where the bipyridines interact among each other by edge‐to‐face C−H⋅⋅⋅π interactions (C⋅⋅⋅π 3.57 Å, N1⋅⋅⋅N2 5.42 Å). The B(OH)_2_ groups are twisted out‐of‐plane of the aromatic ring (O1−B1−C1−C3, −29.2(2)°; O1−B1−C1−C2, 150.05(15)°). The resulting 2D framework is further stabilized by weaker C−H⋅⋅⋅O(B) (C⋅⋅⋅O, 3.223(2) Å; ∠C−H⋅⋅⋅O, 133°) interactions (Figure 2b). The layers interact by C−H⋅⋅⋅π (C⋅⋅⋅π 2.89 Å) interactions among 1,4‐bdba and bpeta, which drives the formation of a 3D supramolecular network (Figure 2c).

### Assemblies of 1,3‐bdba and water‐assisted hydrogen‐bonding

The solid‐state structures of [(1,3‐bdba)(bpe)_2_(H_2_O)_2_] (3) and [(1,3‐bdba)(bpeta)_2_(H_2_O)] (4) involve the generation of supramolecular catemers, in which included water molecules extend the hydrogen‐bonding motifs. The role of water to organize molecules in cocrystals of diboronic acids has been identified first experimentally,[^22^, ^23^] with a theoretical treatment suggesting significant binding energies.^[30]^

**Figure 3:**
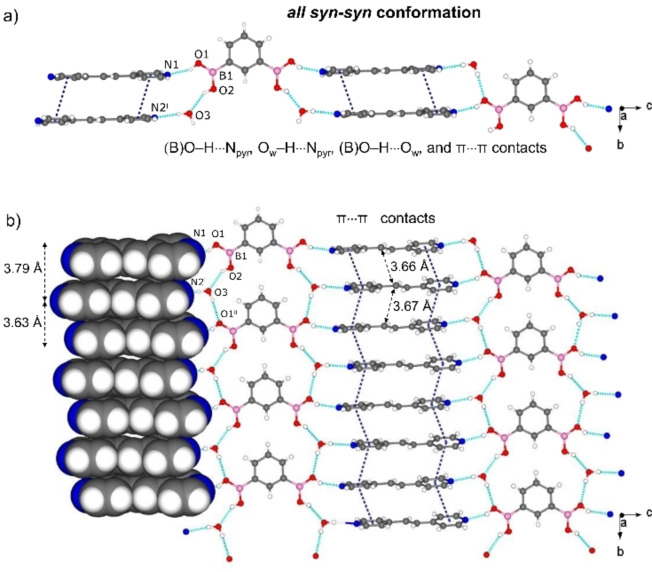
X‐ray structure of 3: a) catemer sustained by (B)O−H⋅⋅⋅N_pyr_, O_w_−H⋅⋅⋅N_pyr_, (B)O−H⋅⋅⋅O_w_, and π⋅⋅⋅π interactions, b) 2D hydrogen‐bonded framework in *bc*‐plane as result of O_w_−H⋅⋅⋅O(B) and additional π⋅⋅⋅π contacts. Symmetry operators: i) 0.5−*x*, 0.5+*y*, 0.5−*z*, (ii) *x*, 1+*y*, *z*.

The components of 3 crystallize in the monoclinic space group *C2/c*. The asymmetric unit consists of one molecule of bpe, a half molecule of 1,3‐bdba and one molecule of water (Figure S4, see Supporting Information), indicating a 1 : 2 : 2 stoichiometry. A single B(OH) group participates in a (B)O−H⋅⋅⋅N_pyr_ [O⋅⋅⋅N, 2.676(4) Å; ∠O−H⋅⋅⋅N, 152°] hydrogen bond. Inclusion of the water results in a highly organized 2D framework sustained by O_w_−H⋅⋅⋅N_pyr_ [O⋅⋅⋅N, 2.831(4) Å; ∠O−H⋅⋅⋅N, 169°] and (B)O−H⋅⋅⋅O_w_/O_w_−H⋅⋅⋅O(B) [O⋅⋅⋅O, 2.793 (4) Å/3.028(4) Å; ∠O−H⋅⋅⋅O, 144/171°] hydrogen bonds. The packing is isostructural to the hydrogen‐bonded analog [(1,3‐bdba)(bpy)_2_(H_2_O)_2_].^[22]^ The acid adopts an *all syn‐syn* conformation with the B(OH)_2_ groups approximately coplanar with the aromatic ring (distortion angle: −3.2(5)° for C2−C1−B1−O1). The pyridyl rings of bpe deviate from coplanarity (twist angle ∼9.8°). As a consequence of the assembly, the bpe molecules form infinite face‐to‐face π⋅⋅⋅π stacks with nearest‐neighbor C=C bonds separated on the order of 3.67 Å, which satisfies the geometry criteria for a [2+2] photodimerization^[27]^ (Figure 3a and b). When irradiated with UV‐radiation, the cocrystal 3 was determined to be photoactive to generate tpcb in quantitative yield (Figure S22; see Supporting Information). We note that after the photoreaction, the PXRD diffractogram exhibited significant broadening (Figure S14; see Supporting Information) excluding a SCSC transformation in this case.

**Figure 4:**
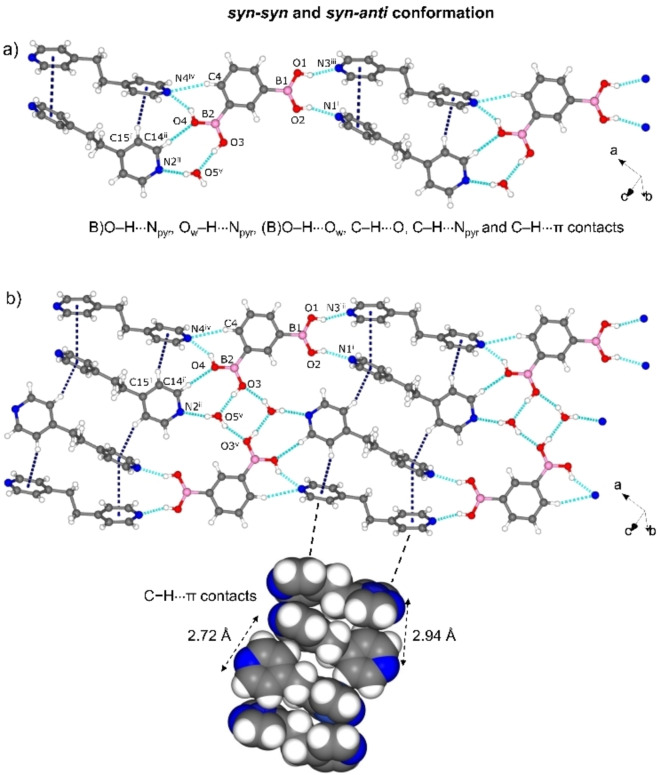
X‐ray structure of 4: a) catemer sustained by (B)O−H⋅⋅⋅N_pyr_, O_w_−H⋅⋅⋅N_pyr_, (B)O−H⋅⋅⋅O_w_, C−H⋅⋅⋅O, C−H⋅⋅⋅N_pyr_ and, C−H⋅⋅⋅π contacts, b) 2D hydrogen‐bonded framework based on O_w_−H⋅⋅⋅O and additional C−H⋅⋅⋅π contacts in *ab*‐plane. Symmetry operators: i) −*x*, 1−*y*,1−*z*; ii) 2−*x*, 1−*y*, 2−*z*; iii) −*x*, −*y*, 1−*z*; iv) 2−*x*, −*y*, 2−*z*; v) 1−*x*, 1−*y*, 2−*z*.

The components of 4 crystallize in the triclinic space group *P‐1* with one 1,3‐bdba, two bpeta, and a water molecule in the asymmetric unit (Figure S5, see Supporting Information) yielding now a 1 : 2 : 1 composition. The (B)OH groups interact with three bpeta molecules by three (B)O−H⋅⋅⋅N_pyr_ (O⋅⋅⋅N, 2.743(2) Å/2.747(1) Å/2.845(2) Å; ∠O−H⋅⋅⋅N, 145°/156°/156°) hydrogen bonds. In the arrangement, one of the acid groups serves as a DD hydrogen‐bond donor module. The remaining (B)OH group participates in both (B)O−H⋅⋅⋅O_w_ (O⋅⋅⋅O, 2.721(2) Å; ∠O−H⋅⋅⋅O, 169°] and O_w_−H⋅⋅⋅O(B) [O⋅⋅⋅O, 2.771(2) Å; ∠O−H⋅⋅⋅O, 160°) hydrogen bonds with the included water (Figure 4a). The water molecule also interacts with the bipyridine by O_w_−H⋅⋅⋅N_pyr_ (O⋅⋅⋅N, 2.844(2) Å; ∠O−H⋅⋅⋅N, 164°) hydrogen bonds. The acid exhibits both *syn‐syn* and *syn‐anti* conformations. The B(OH)_2_ groups in 1,3‐bdba are approximately coplanar to the aromatic ring [torsion angles: −2.5(2)° to −4.3(2)°] while the pyridyl groups of the two bipyridines are significantly twisted (torsion angles: 46° and 88°). The components assemble to generate a 2D structure stabilized by C−H⋅⋅⋅N_pyr_ and C−H⋅⋅⋅O contacts within the *ab*‐plane, with the bpeta molecules engaged in edge‐to‐face π⋅⋅⋅π (3.91 Å) and C−H⋅⋅⋅π (2.72–2.94 Å) interactions (Figure 4b).

### Assemblies of 4,4'‐bphdba and in‐situ linker reaction

The solid‐state structures of [(4,4'‐bphdba)(bpe)] (5) and [(4,4'‐bphba‐me)(bpeta)] (6) involve, similar to 1 and 2, the generation of 1D hydrogen‐bonded arrays sustained by B(O)−H⋅⋅⋅N_pyr_ hydrogen bonds. For 5, the acid conformation results in a 2D hydrogen‐bonded framework. For 6, an in‐situ linker reaction with methanol to generate a hemiester effectively restricts the self‐assembly process to a single dimension.

**Figure 5:**
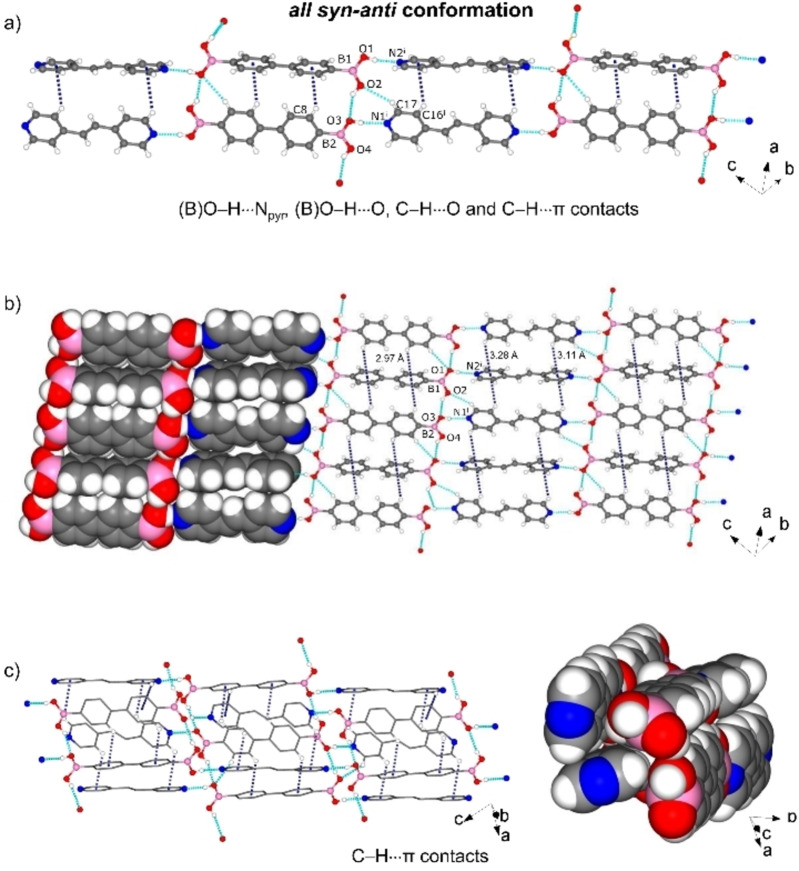
X‐ray structure of 5: a) 1D structural motif formed by (B)O−H⋅⋅⋅N_pyr_, (B)O−H⋅⋅⋅O, C−H⋅⋅⋅O and C−H⋅⋅⋅π contacts, b) 2D hydrogen bonded framework in the *bc*‐plane, c) 3D network through C−H⋅⋅⋅π contacts. Symmetry operator: i) −*x*, 2−*y*,1−*z*.

The components of 5 assemble in the triclinic space group *P‐1*. The asymmetric unit contains two halves each of 4,4'‐bphdba and bpe molecules (Figure S6, see Supporting Information) that interact by B(O)−H⋅⋅⋅N_pyr_ [O⋅⋅⋅N, 2.718(2) Å/2.763(2) Å; ∠O−H⋅⋅⋅N, 171°/172°] and B(O)−H⋅⋅⋅O [O⋅⋅⋅O, 2.959(2) Å/2.865(2) Å; ∠O−H⋅⋅⋅O, 130°/150°] hydrogen bonds. In contrast to cocrystals 1–4, the diboronic acid‐coformer stoichiometry is now 1 : 1 instead of 1 : 2. In the 1 : 2 cocrystals, all B−OH hydrogens are involved in hydrogen bonds with the bipyridines either through direct contacts [(B)O−H⋅⋅⋅N_pyr_] or a water‐expanded motif as observed in 3 and 4. In 5, half the B−OH hydrogens form B(O)−H⋅⋅⋅N_pyr_ hydrogen bonds whilst the remaining are involved in B(O)−H⋅⋅⋅O(B) bridges, generating infinite 1D strands of 4,4'‐bphdba. In 5, both acid groups exhibit an *all syn‐anti* conformation (Figure 5a). One B(OH)_2_ group is coplanar with the aromatic ring (distortion angles: −0.2(2)° for C12−C7−B2−O4 and −1.2(2)° for C8‐C7‐B2‐O3), whereas the second is out‐of‐plane (distortion angles: 44.0(2)° for C6−C1−B1−O1 and 44.4(2)° for C2−C1−B1−O2). The pyridyl rings of both molecules of bpe are coplanar as imposed by the crystallographic symmetry (twist angle=0.00°).

**Scheme 3:**
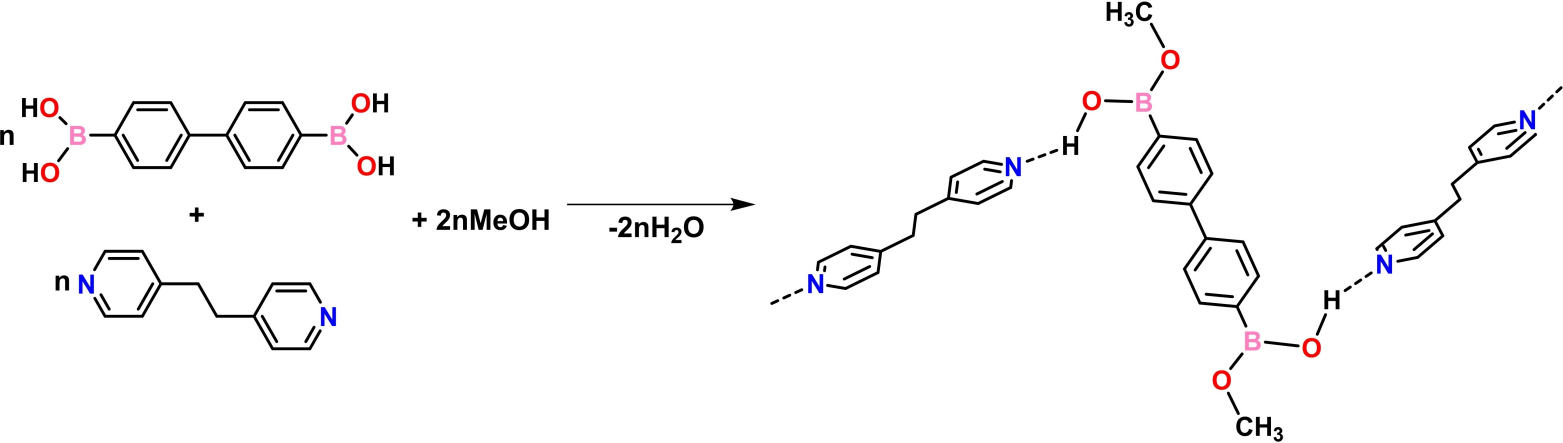
Transformation of 4,4'‐bphdba to the hemiester‐based cocrystal 6. Reaction involves partial replacement of (B)OH by −OMe and loss of water.

**Figure 6:**
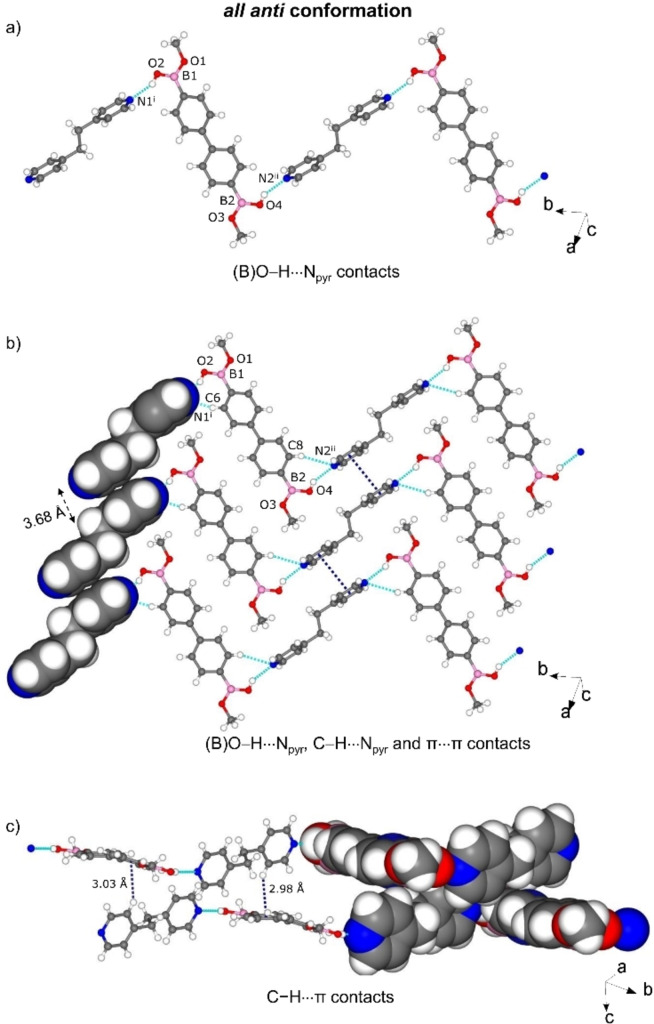
X‐ray structure of 6: a) 1D motif formed by (B)O−H⋅⋅⋅N_pyr_ hydrogen bonds, b) 2D hydrogen bonded framework in the *bc*‐plane, c) C−H⋅⋅⋅π contacts between the 4,4'‐bphdba and bpeta along *c*. Symmetry operators: i) 1−*x*, 2−*y*, −*z*; ii) −*x*, 1−*y*, 1−*z*.

The packing is further supported by C−H⋅⋅⋅O and C−H⋅⋅⋅π interactions, giving a 2D arrangement in the *bc*‐plane (Figure 5b). Different from cocrystals 1–4, both the 4,4'‐bphdba and bpe molecules are packed into infinite columns by π‐stacking through C−H⋅⋅⋅π contacts (C⋅⋅⋅π 2.97 Å for 4,4'‐bphdba and C⋅⋅⋅π 3.11‐3.28 Å for bpe), with adjacent 4,4'‐bphdba and bpe entities being approximately orthogonal (4,4'‐bphdba, 89°; bpe, 64°). C−H⋅⋅⋅π contacts (C⋅⋅⋅π 2.82–3.16 Å) are also present between the 2D layers to give an overall 3D network (Figure 5c). The components of 6, as with 5, assemble in the triclinic space group *P‐1*. The asymmetric unit contains one 4,4'‐bphdba‐me and two halves of bpeta molecules (Figure S7, see Supporting Information). The formation of 4,4'‐bphdba‐me presumably occurred as base‐catalyzed boronic acid methanolysis^[31]^ (Scheme 3). The resulting hemiester serves as a restricted DD’ hydrogen‐bond‐donor, enabling the rupture of the tight hydrogen bonding network observed in compounds 1–4 and assembling with bpeta to generate 1D zig‐zag chains sustained by (B)−O⋅⋅⋅N_pyr_ [O⋅⋅⋅N, 2.771(2) Å/2.787(2) Å; ∠O−H⋅⋅⋅N, 152°/150°] and weaker C−H⋅⋅⋅N_pyr_ hydrogen bonds. The hemiester adopts an *all‐anti* conformation (Figure 6a), with the B(OH)Me groups being approximately coplanar to the aromatic ring (distortion angles: 4.6(2)° for C2−C1−B1−O1 and 8.9(2)° for C8−C7−B2−O4). The chains exhibit a tongue‐in‐groove fit to form a 2D herringbone‐type architecture characterized by face‐to‐face π⋅⋅⋅π stacks (3.68 Å) of bpeta molecules in the crystallographic *bc*‐plane (Figure 6b).

The 4,4'‐bphdba and bpeta components also interact by C−H⋅⋅⋅π contacts (C⋅⋅⋅π 2.981–3.034 Å) along *c* (Figure 6c). We are unaware of a case wherein a boronic acid has undergone an in‐situ transformation to form a cocrystal. The in‐situ reaction effectively serves to restrict the dimensionality of the self‐assembly process relative to 5. We note that in‐situ linker transformations are reported for metal–organic frameworks (i. e., MOFs) and materials.^[32]^ The one‐pot reactions have been shown to be useful to generate unusual organic ligands that are a challenge to access in the generation of novel MOF materials. In the current study, the in‐situ formation of the hemiester may provide a unique avenue to construct novel organic cocrystals based on diboronic acids.

## Conclusion

We have reported a comparison of structural and reactivity aspects concerning cocrystals and hydrogen‐bonded frameworks involving diboronic acids and bipyridines. The comparative analysis reveals that the coformers with the diboronic acids play an important role to support adaptability of the diboronic acids in the self‐assembly process. UV radiation of single crystals of 1 resulted in the 1D‐to‐2D crystal‐to‐crystal [2+2] photodimerization. Our work also shows the formation of hemiester 6 by in‐situ linker transformation. We expect our results to impact the further development of polymeric assemblies based on boronic acids in organic solids and as related to field of crystal engineering.

Deposition Number(s) 2121702 (for 1), 2121707 (for 1R), 2121704 (for 2), 2121706 (for 3), 2121705 (for 4), 2121703 (for 5), 2121701 (for 6) contain(s) the supplementary crystallographic data for this paper. These data are provided free of charge by the joint Cambridge Crystallographic Data Centre and Fachinformationszentrum Karlsruhe Access Structures service.

## Conflict of interest

The authors declare no conflict of interest.

1

## Supporting information

As a service to our authors and readers, this journal provides supporting information supplied by the authors. Such materials are peer reviewed and may be re‐organized for online delivery, but are not copy‐edited or typeset. Technical support issues arising from supporting information (other than missing files) should be addressed to the authors.

Supporting Information

## Data Availability

The data that support the findings of this study are available in the supplementary material of this article.
