# Supplementary material for: Structures and Reactivities of Cocrystals Involving Diboronic Acids and Bipyridines: In Situ Linker Reaction and 1D‐to‐2D Dimensionality Change via Crystal‐to‐Crystal Photodimerization
Source: Chemistry. 2022 Mar 25;28(25):e202104604. doi: 10.1002/chem.202104604 (PMC9310599; doi:10.1002/chem.202104604)
Supplement: Supplementary file 1 — Supporting Information [file CHEM-28-0-s001.pdf]

Chemistry–A European Journal

Supporting Information

Structures and Reactivities of Cocrystals Involving Diboronic Acids and Bipyridines: In Situ Linker Reaction and 1D-to-2D Dimensionality Change via Crystal-to-Crystal Photodimerization

María Guadalupe Vasquez-Ríos , Gonzalo Campillo-Alvarado, Dale C. Swenson, Herbert Höpfl,* and Leonard R. MacGillivray*

S1. Experimental section

S2. Single-crystal X-ray diffraction data

S3. Hydrogen-bond table

S4. Powder X-ray diffraction data

S5. NMR spectral data

S6. References

S1. Experimental section

Reagents. 1,2-bis(4-pyridyl)ethane (bpeta), 1,2-bis(4-pyridyl)ethylene (bpe), 1,3-benzendiboronic acid (1,3-bdba), 1,4-benzendiboronic acid (1,4-bdba), 4,4´-biphenyldiboronic acid (4,4´-bphdba), methanol, acetone and water were obtained from Sigma-Aldrich and used as received without further purification.

Instrumental. Single-crystal X-ray diffraction (SCXRD) studies for 1-6, and 1R were performed on a Bruker Nonius APEX II Kappa instrument using Mo-K_{α} (λ = 0.71073 Å) radiation. For 1, 2, 3, and 6, frames were collected at T = 298.15 K, but for compounds 1R, 4, and 5 the crystals were not stable at that temperature and were collected at T = 150.15 K. Crystals were mounted in Paratone oil on a Mitagen magnetic mount. Corrections were made for Lorentz and polarization effects and the programs in the APEXII package were used for data reduction. Structure solution, refinement, and data output were performed with the OLEX[1] program package using SHEXLTL[2] for the structure solution and SHELXL-2014[3] for the refinement. Non-hydrogen atoms were refined anisotropically. Hydrogen atoms were placed in geometrically calculated positions using the riding model. For the refinement of the disordered bpe ligand and tpcb molecules, geometry and U_{ij} restraints were used.[3] Diamond was used for the creation of figures.[4] Crystallographic data for the structures reported in this paper have been deposited with the Cambridge Crystallographic Data Centre as supplementary publications no. CCDC-2121701–2121707. Copies of the data can be obtained free of charge on application to CCDC, 12 Union Road, Cambridge CB2 1EZ, UK (fax: (+44)1223-336-033; e-mail: deposit@ccdc.cam.ac.uk, www: http://www.ccdc.cam.ac.uk).

Powder X-ray diffraction (PXRD) analyses were collected on samples mounted on glass slides using a Bruker D8 Avance X-ray diffractometer with Cu-K_{α} radiation (1.54184 Å). The equipment was operated at 40 kV and 30 mA, and the data were collected at room temperature in the range 2θ = 5-40°. NMR studies were carried out with a Bruker AVANCE 500 instrument using DMSO-d_6 as the NMR solvent. Chemical shifts are expressed in parts per million.

Syntheses

[(1,4-bdba)$(bpe)_2$] (1). 1,4-bdba (0.020 g, 0.121 mmol) and bpe (0.044, 0.241 mmol) were dissolved in 2 mL of methanol, and a solution was stirred for 10 minutes at room temperature. After three days, colorless plate crystals suitable for SCXRD

analysis were formed. ^{1}H NMR (500 MHz, DMSO-d$_6$): δ = 7.54 (s, 4H, Hd), 7.61 (s, 8H, Hc), 7.74 (s, 4H, Ha), 8.00 (s, 4H, (BOH)$_2$), 8.61 (s, 8H, Hb) ppm.

[(1,4-bdba)(bpeta)$_2$] (2). 1,4-bdba (0.010 g, 0.060 mmol) was added to a solution of bpeta (0.022, 0.121 mmol) in 2 mL of methanol. The solution was stirred for 10 minutes at room temperature. After one day colorless lath-like crystals had formed by slow solvent evaporation, which were suitable for SCXRD analysis. ^{1}H NMR (500 MHz, DMSO-d$_6$): δ = 2.94 (s, 8H, Hd), 7.26 (d, 8H, ^3J = 1.3, Hc), 7.75 (s, 4H, Ha), 8.05 (s, 4H, B(OH)$_2$), 8.45 (dd, 8H, ^3J = 4.5, 1.4 Hz, Hb) ppm.

[(1,3-bdba)(bpe)$_2$(H$_2$O)$_2$] (3). A solution of 1,3-bdba (0.020 g, 0.121 mmol) and bpe (0.044 g, 0.241 mmol) in 3 mL of acetone was stirred for 10 minutes at room temperature. Colorless prism-like crystals were formed after 7 days, which were suitable for SCXRD analysis. Several attempts were made to recrystallize 3 after photoreaction but not suitable crystals for SCXRD analysis were obtained. ^{1}H NMR (500 MHz, DMSO-d$_6$): δ = 7.30 (t, ^3J = 7.4 Hz, 1H, Hc), 7.62 (m, 8H, He), 7.54 (s, 4H, Hf), 7.83 (dd, ^{3}J = 7.4, 1.3 Hz, 2H, Hb), 8.00 (s, 4H, B(OH)$_2$), 8.24 (s, 1H, Ha), 8.61 (s, 8H, Hd) ppm.

[(1,3-bdba)(bpeta)$_2$(H$_2$O)] (4). A solution of 1,3-bdba (0.010 g, 0.060 mmol) and bpeta (0.022, 0.121 mmol) in 3 mL of acetone was stirred for 10 minutes at room temperature. Colorless irregular prism-like crystals were formed after 5 days, which were suitable for SCXRD analysis. ^{1}H NMR (500 MHz, DMSO-d$_6$): δ = 2.94 (s, 8H, Hf), 7.26 (m, 8H, He), 7.29 (m, 1H, Hc), 7.81 (dd, 2H, ^3J = 7.4, 1.3 Hz, Hb), 7.97 (s, 4H, B(OH)$_2$), 8.22 (s, 1H, Ha), 8.45 (s, 8H, Hd) ppm.

[(4,4´-bphdba)(bpe)] (5). 4,4´-bphdba (0.010 g, 0.041 mmol) was dissolved in 3 mL of methanol, and bpe (0.008, 0.041 mmol) was added. The solution was stirred at room temperature for 10 minutes. After five days, colorless lath-like crystals suitable for SCXRD analysis were formed by slow solvent evaporation. ^{1}H NMR (500 MHz, DMSO-d$_6$): δ = 7.54 (s, 2H, He), 7.62 (dd, ^3J = 4.6, 1.5 Hz, 4H, Hd), 7.67 (d, ^3J = 8.2 Hz, 4H, Hb), 7.90 (d, ^3J = 8.2 Hz, 4H, Ha), 8.07 (s, 4H, B(OH)$_2$), 8.61 (dd, ^3J = 4.6, 1.4 Hz, 4H, Hc) ppm.

[(4,4´-bphdba-me)(bpeta)] (6). A solution of 4,4´-bphdba (0.010 g, 0.041 mmol) and bpeta (0.008, 0.041 mmol) in 2 mL of methanol was stirred for 10 minutes at room temperature, giving after four days colorless rod-like crystals suitable for SCXRD analysis. ^{1}H NMR (500 MHz, DMSO-d$_6$): δ = 2.95 (s, 4H, He), 7.26 (dd, ^3J = 1.5 Hz, 4H, Hd), 7.67 (d, ^3J = 8.2 Hz, 4H, Hb), 7.89 (d, ^3J = 8.1 Hz, 4H, Ha), 8.07 (s, 4H, B(OH)$_2$), 8.45 (dd, ^3J = 1.6 Hz, 4H, Hc) ppm.

S2. Single-crystal X-ray diffraction data

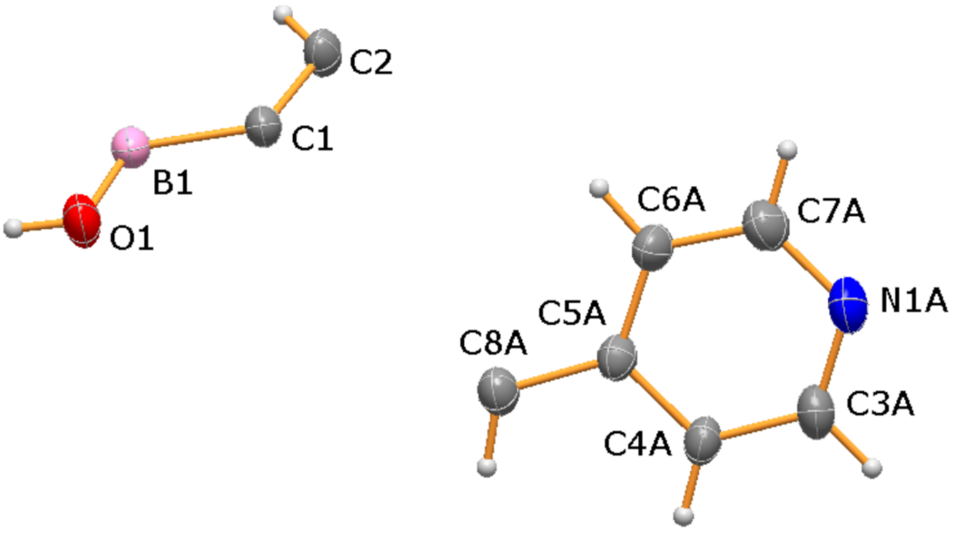

Figure S1. Perspective view of asymmetric unit of compound 1. Displacement ellipsoids shown at the 30% probability level. For clarity, disorder of bpe is not shown.

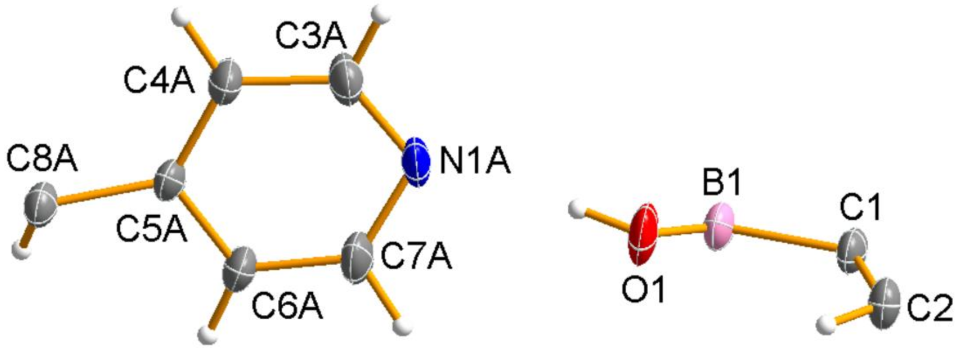

Figure S2. Perspective view of asymmetric unit of 1R. Displacement ellipsoids shown at the 30% probability level. For clarity, disorder of tpcb is not shown.

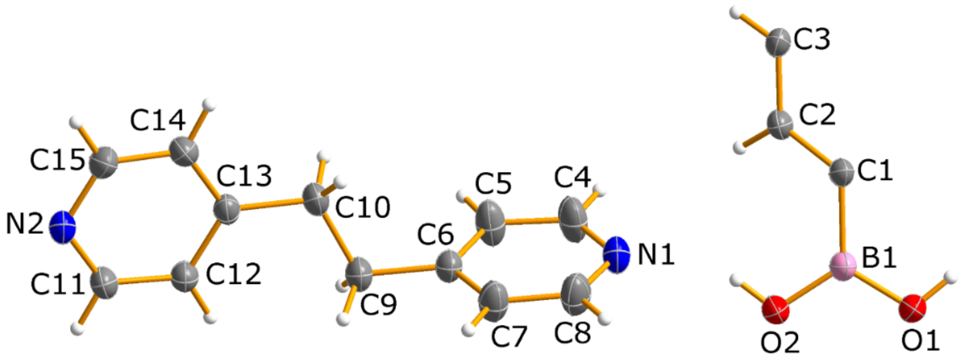

Figure S3. Perspective view of asymmetric unit of 2. Displacement ellipsoids shown at the 50% probability level.

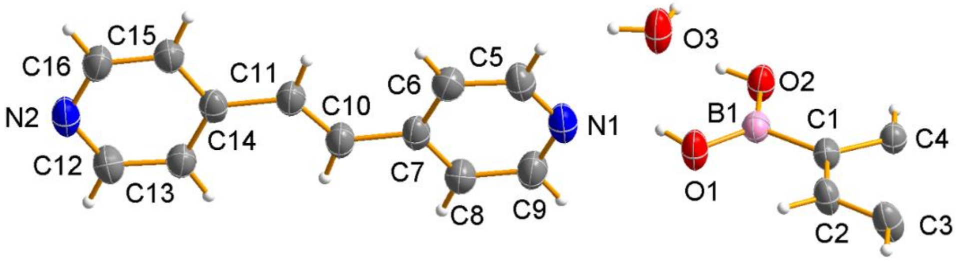

Figure S4. Perspective view of asymmetric unit of 3. Displacement ellipsoids shown at the 30% probability level.

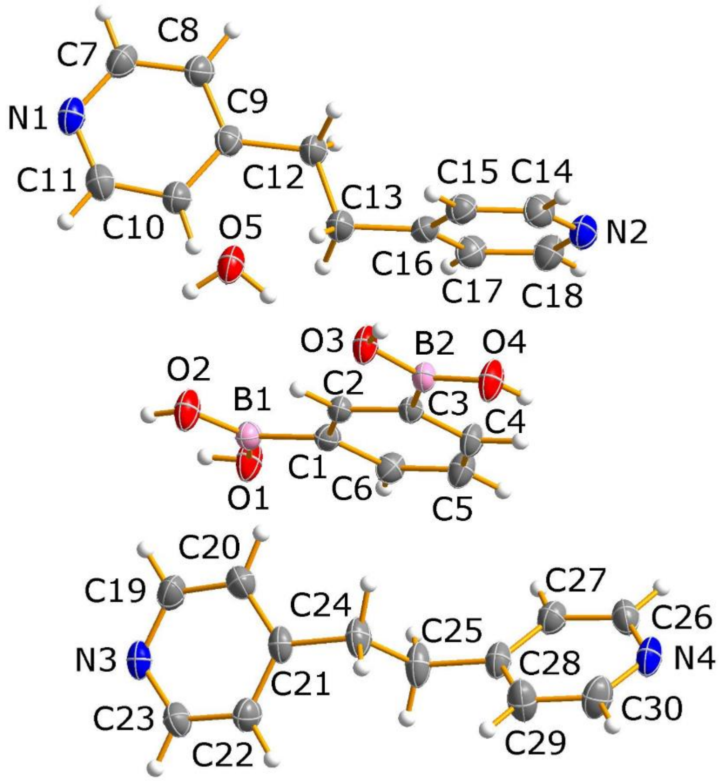

Figure S5. Perspective view of asymmetric unit of 4. Displacement ellipsoids shown at the 50% probability level.

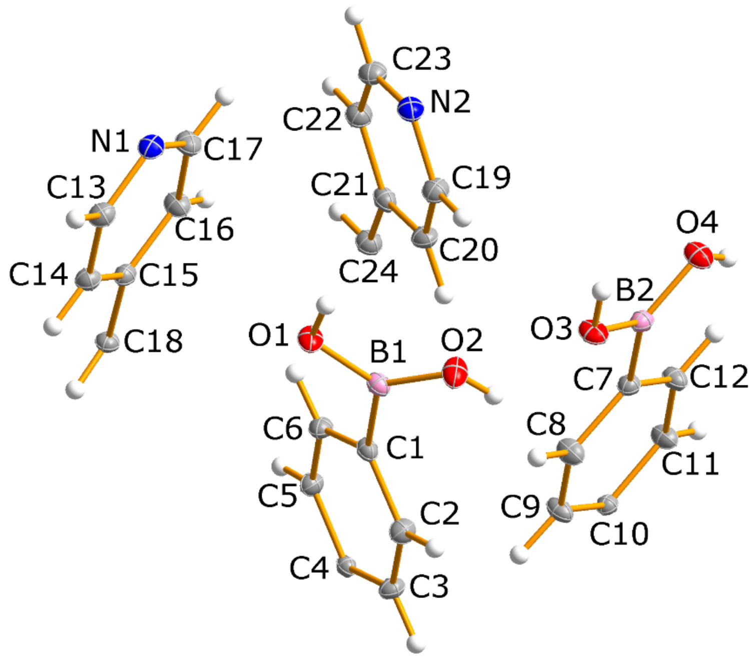

Figure S6. Perspective view of asymmetric unit of 5. Displacement ellipsoids shown at the 30% probability level.

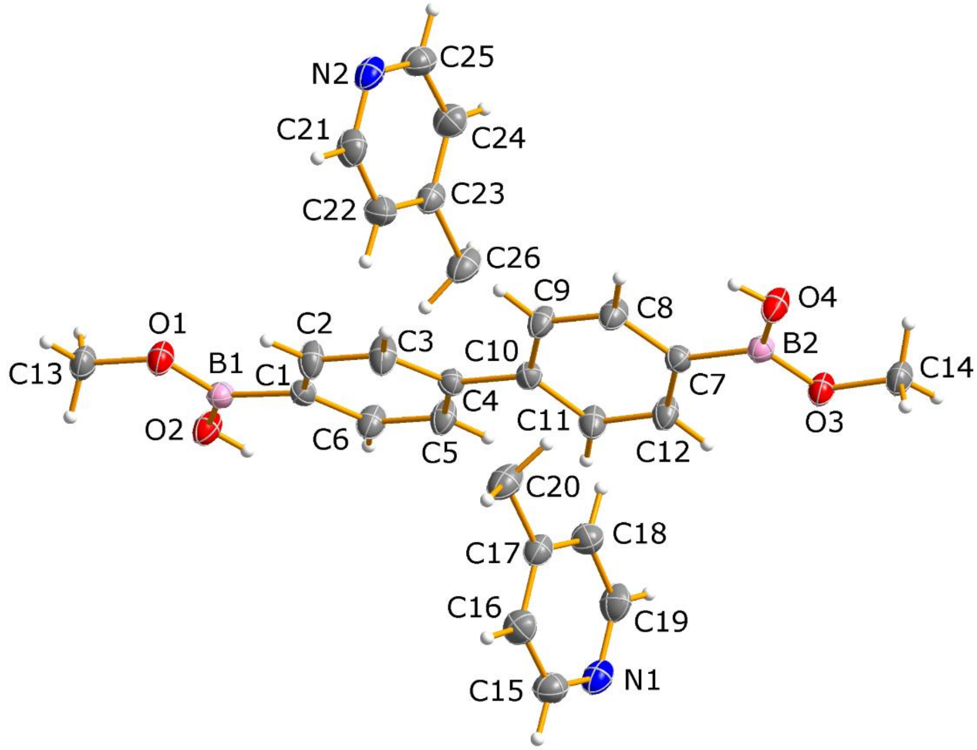

Figure S7. Perspective view of asymmetric unit of 6. Displacement ellipsoids were drawn at 50% probability level.

Table S1. Crystal data and structure refinement data for 1.

Formula	$C_{30}H_{28}B_2N_4O_4$
MW (g mol^{-1})	530.18
T (K)	298.15
Space group	*C2/m*
Radiation	Mo K_{α}
a (Å)	9.0736(9)
b (Å)	22.140(2)
c (Å)	6.8746(7)
α (deg)	90
β (deg)	105.148(5)
γ (deg)	90
V (Å^3)	1333.0(2)
Z	2
μ (mm^{-1})	0.088
ρ_{calcd} (g cm^{-3})	1.321
F(000)	556.0
Crystal size/mm^3	0.18 × 0.13 × 0.045
Θ Range/°	3.68 to 50.708
Limiting indices	-10 ≤ h ≤ 10<br>-26 ≤ k ≤ 22<br>-8 ≤ 1 ≤ 8
Reflections collected	3811
Independent reflections	1261 [R_{int} = 0.0491, R_{sigma} = 0.0517]
No.data/restrains/parameters	1261/105/156
Goodness of fit on F2	1.024
Final R indexes [I>=2σ(I)]	R_1 = 0.0410, wR_2 = 0.0912
Final R indexes [all data]	R_1 = 0.1070, wR_2 = 0.1156
Largest diff. peak/hole/ e Å^{-3}	0.14/-0.16
CCDC	2121702

Table S2. Crystal data and structure refinement data for 1R.

Formula	$C_{30}H_{28}B_2N_4O_4$
MW (g mol^{-1})	530.18
T (K)	150.15
Space group	*C2/m*
Radiation	Mo K_{α}
a (Å)	9.1318(9)
b (Å)	21.760(2)
c (Å)	7.3373(7)
α (deg)	90
β (deg)	111.610(5)
γ (deg)	90
V (Å^3)	1355.5(2)
Z	2
μ (mm^{-1})	0.086
ρ_{calcd} (g cm^{-3})	1.299
F(000)	556
Crystal size/mm^3	0.175 × 0.13 × 0.015
Θ Range/°	5.15 to 52.744
Limiting indices	-11 ≤ h ≤ 11
	-27 ≤ k ≤ 26
	-9 ≤ 1 ≤ 9
Reflections collected	15573
Independent reflections	1431 [R_{int} = 0.0445, R_{sigma} = 0.0303]
No.data/restrains/parameters	1431/104/162
Goodness of fit on F2	1.081
Final R indexes [I>=2σ(I)]	R_1 = 0.0507, wR_2 = 0.1472
Final R indexes [all data]	R_1 = 0.0637, wR_2 = 0.1567
Largest diff. peak/hole/ e Å^{-3}	0.30/-0.20
CCDC	2121707

Table S3. Crystal data and structure refinement data for 2.

Formula	$C_{15}H_{16}BN_2O_2$
MW (g mol^{-1})	267.11
T (K)	298.15
Space group	*P-1*
Radiation	Mo K_α
a (Å)	8.8956(9)
b (Å)	9.6081(10)
c (Å)	10.2402(10)
α (deg)	98.549(5)
β (deg)	107.012(5)
γ (deg)	117.225(5)
V (Å^3)	701.94(13)
Z	2
μ (mm^{-1})	0.084
ρ_{calcd} (g cm^{-3})	1.264
F(000)	282
Crystal size/mm^3	0.225 × 0.12 × 0.045
Θ Range/°	4.41 to 52.756
Limiting indices	-11 ≤ h ≤ 11
	-10 ≤ k ≤ 12
	-12 ≤ 1 ≤ 12
Reflections collected	13133
Independent reflections	2863 [R_{int} = 0.0293, R_{sigma} = 0.0226]
No.data/restrains/parameters	2863/0/183
Goodness of fit on F2	1.044
Final R indexes [I>=2σ(I)]	R_1 = 0.0419, wR_2 = 0.1074
Final R indexes [all data]	R_1 = 0.0537, wR_2 = 0.1169
Largest diff. peak/hole/ e Å^{-3}	0.18/-0.18
CCDC	2121704

Table S4. Crystal data and structure refinement data for 3.

Formula	$C_{15}H_{16}BN_2O_3$
MW (g mol^{-1})	283.11
T (K)	298.15
Space group	*C2/c*
Radiation	Mo K_{α}
a (Å)	18.341(7)
b (Å)	6.938(3)
c (Å)	24.074(9)
α (deg)	90
β (deg)	107.671(8)
γ (deg)	90
V (Å^3)	2919(2)
Z	8
μ (mm^{-1})	0.089
ρ_{calcd} (g cm^{-3})	1.288
F(000)	1192
Crystal size/mm^3	0.065 × 0.06 × 0.05
Θ Range/°	4.662 to 52.692
Limiting indices	-22 ≤ h ≤ 22
	-8 ≤ k ≤ 8
	-30 ≤ 1 ≤ 28
Reflections collected	33255
Independent reflections	2982 [R_{int} = 0.0612, R_{sigma} = 0.0620]
No.data/restrains/parameters	2982/0/196
Goodness of fit on F2	1.047
Final R indexes [I>=2σ(I)]	R_1 = 0.0768, wR_2 = 0.2216
Final R indexes [all data]	R_1 = 0.1238, wR_2 = 0.2489
Largest diff. peak/hole/ e Å^{-3}	0.22/-0.25
CCDC	2121706

Table S5. Crystal data and structure refinement data for 4.

Formula	$C_{30}H_{34}B_2N_4O_5$
MW (g mol^{-1})	552.23
T (K)	150.15
Space group	*P-1*
Radiation	Mo K_α
a (Å)	9.4058(9)
b (Å)	11.0681(11)
c (Å)	14.9534(15)
α (deg)	94.424(5)
β (deg)	107.269(5)
γ (deg)	95.548(5)
V (Å^3)	1470.4(3)
Z	2
μ (mm^{-1})	0.084
ρ_{calcd} (g cm^{-3})	1.247
F(000)	584
Crystal size/mm^3	0.16 × 0.1 × 0.09
Θ Range/°	4.438 to 52.776
Limiting indices	-11 ≤ h ≤ 11
	-13 ≤ k ≤ 13
	-18 ≤ 1 ≤ 17
Reflections collected	24484
Independent reflections	6001 [R_{int} = 0.0333, R_{sigma} = 0.0272]
No.data/restrains/parameters	6001/0/377
Goodness of fit on F2	1.026
Final R indexes [I>=2σ(I)]	R_1 = 0.0395, wR_2 = 0.0907
Final R indexes [all data]	R_1 = 0.0532, wR_2 = 0.1022
Largest diff. peak/hole/ e Å^{-3}	0.28/-0.24
CCDC	2121705

Table S6. Crystal data and structure refinement data for 5.

Formula	$C_{24}H_{22}B_2N_2O_4$
MW (g mol^{-1})	424.05
T (K)	150.15
Space group	*P-1*
Radiation	Mo K_α
a (Å)	9.6343(10)
b (Å)	10.6076(11)
c (Å)	12.2331(12)
α (deg)	87.325(5)
β (deg)	69.488(5)
γ (deg)	63.302(5)
V (Å^3)	1036.88(19)
Z	2
μ (mm^{-1})	0.091
ρ_{calcd} (g cm^{-3})	1.358
F(000)	444
Crystal size/mm^3	0.13 × 0.055 × 0.025
Θ Range/°	4.336 to 52.8
Limiting indices	-12 ≤ h ≤ 11
	-13 ≤ k ≤ 1
	-15 ≤ 1 ≤ 15
Reflections collected	19966
Independent reflections	4251 [R_{int} = 0.0353, R_{sigma} = 0.0276]
No.data/restrains/parameters	4251/0/293
Goodness of fit on F2	1.029
Final R indexes [I>=2σ(I)]	R_1 = 0.0417, wR_2 = 0.1081
Final R indexes [all data]	R_1 = 0.0565, wR_2 = 0.1176
Largest diff. peak/hole/ e Å^{-3}	0.34/-0.23
CCDC	2121703

Table S7. Crystal data and structure refinement data for 6.

Formula	$C_{26}H_{28}B_2N_2O_4$
MW (g mol^{-1})	454.12
T (K)	298.15
Space group	*P-1*
Radiation	Mo K_{α}
a (Å)	7.8843(2)
b (Å)	12.7325(3)
c (Å)	13.5657(4)
α (deg)	69.0100(10)
β (deg)	73.3440(10)
γ (deg)	76.2670(10)
V (Å^3)	1204.21(6)
Z	2
μ (mm^{-1})	0.083
ρ_{calcd} (g cm^{-3})	1.252
F(000)	480
Crystal size/mm^3	0.18 × 0.08 × 0.065
Θ Range/°	3.97 to 52.856
Limiting indices	-9 ≤ h ≤ 9
	-15 ≤ k ≤ 15
	-16 ≤ 1 ≤ 16
Reflections collected	19022
Independent reflections	4902 [R_{int} = 0.0299, R_{sigma} = 0.0221]
No.data/restrains/parameters	4902/0/311
Goodness of fit on F2	1.056
Final R indexes [I>=2σ(I)]	R_1 = 0.0494, wR_2 = 0.1474
Final R indexes [all data]	R_1 = 0.0594, wR_2 = 0.1576
Largest diff. peak/hole/ e Å^{-3}	0.31/-0.19
CCDC	2121701

S3. Hydrogen-bond table

Table S8. Geometric parameters for intermolecular contacts in the crystal structures of 1-6 and 1R.

Compound	D–H···A [Å]	D–H [Å]	D···A [Å]	H···A [Å]	∠D–H···A (deg)	Symmetry code
1	O1–H1···N1A	0.82	2.735(14)	1.97	155	x, y, z
	O1–H1···N1B	0.82	2.860(12)	2.11	152	x, y, z
1R	O1–H1···N1A	0.84	2.742(15)	1.94	160	x, y, z
	O1–H1···N1B	0.84	2.778(15)	1.99	156	x, y, z
2	O1–H1···N2	0.82	2.821(2)	2.04	158	1+x, 1+y, -1+z
	O2–H2···N1	0.82	2.806(2)	2.04	154	x, y, z
	C11–H11···O1	0.93	3.570(2)	2.67	163	-x, 1-y, 1-z
	C12–H12···O2	0.93	3.223(2)	2.52	133	-x, 1-y, 1-z
3	O1–H1···N1	0.82	2.676(4)	1.92	152	x, y, z
	O2–H2···O3	0.82	2.793(4)	2.08	144	x, y, z
	O3–H3B···N2	0.85	2.831(4)	1.99	169	0.5-x, 0.5+y, 0.5-z
	O3–H3A···O1	0.85	3.028(4)	2.18	171	x, 1+y, z
4	O1–H1···N3	0.84	2.747(1)	1.95	156	-x, -y, 1-z
	O2–H2···N1	0.84	2.845(2)	2.06	156	-x, 1-y, 1-z
	O3–H3···O5	0.84	2.721(2)	1.89	169	1-x, 1-y, 2-z
	O5–H5A···O3	0.87	2.771(2)	1.94	160	x, y, z
	O4–H4···N4	0.84	2.743(2)	2.01	145	2-x, -y, 2-z
	O5–H5B···N2	0.87	2.844(2)	1.99	164	-1+x, y, z
	C6–H6···O1	0.95	2.849(2)	2.48	103	x, y, z
	C4–H4···N4	0.95	3.604(2)	2.68	162	2-x, -y, 2-z
	C14–H14···O4	0.95	3.317(2)	2.49	146	2-x, 1-y, 2-z
	C29–H29···O3	0.95	3.397(2)	2.46	167	1-x, -y, 2-z
5	O1–H1···N2	0.84	2.718(2)	1.88	171	-x, 2-y, 1-z
	O2–H2···O3	0.84	2.959(2)	2.35	130	x, y, z
	O3–H3A···N1	0.84	2.763(2)	1.93	172	-x, 2-y, 1-z
	O4–H4···O1	0.84	2.865(2)	2.11	150	-1+x, y, z
	C12–H12···O1	0.95	3.574(2)	2.67	159	1+x, y, z
	C17–H17···O2	0.95	3.399(2)	2.61	140	-x, 2-y, 1-z
6	O2–H2···N1	0.82	2.771(2)	2.02	152	1-x, 2-y, -z
	O4–H4···N2	0.82	2.787(2)	2.04	150	-x, 1-y, 1-z
	C6–H6···N1	0.93	3.534(2)	2.66	156	1-x, 2-y, -z
	C8–H8···N2	0.93	3.545(2)	2.65	161	-x, 1-y, 1-z

S4. Powder X-ray diffraction data

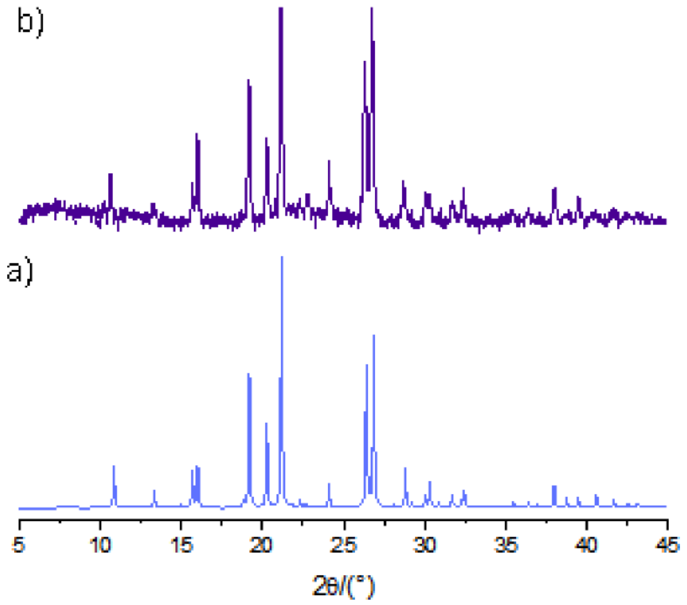

Figure S8. PXRD patterns of 1. a) Calculated and b) experimental.

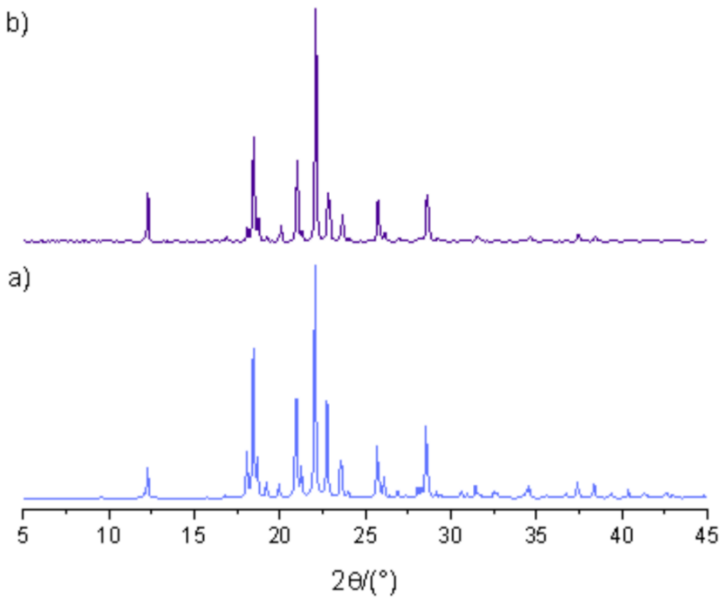

Figure S9. PXRD patterns of 2. a) Calculated and b) experimental.

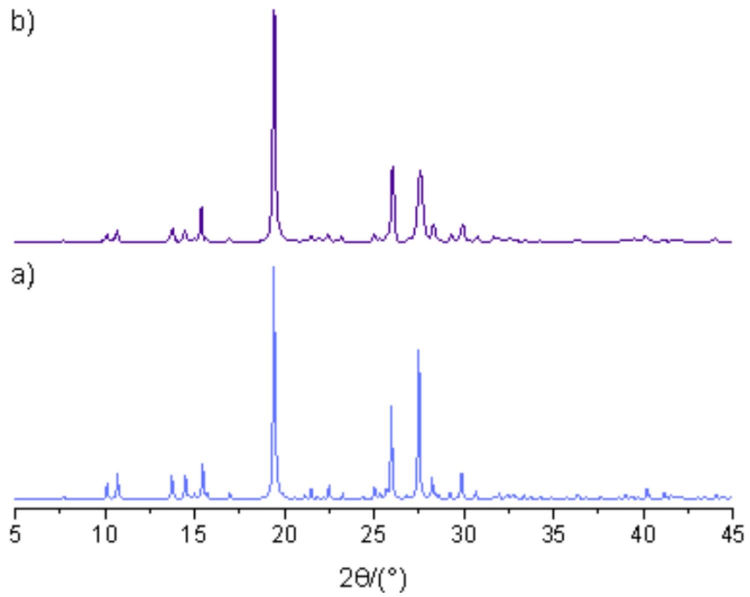

Figure S10. PXRD patterns of 3. a) Calculated and b) experimental.

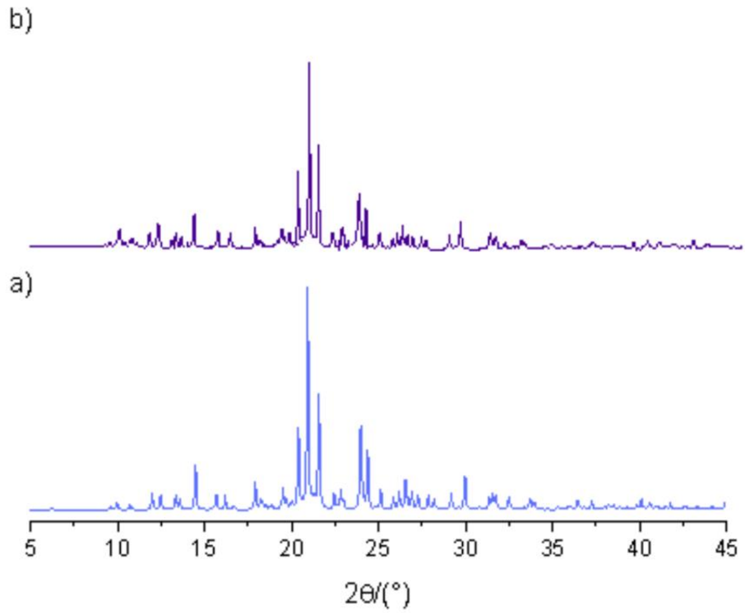

Figure S11. PXRD patterns of 4. a) Calculated and b) experimental.

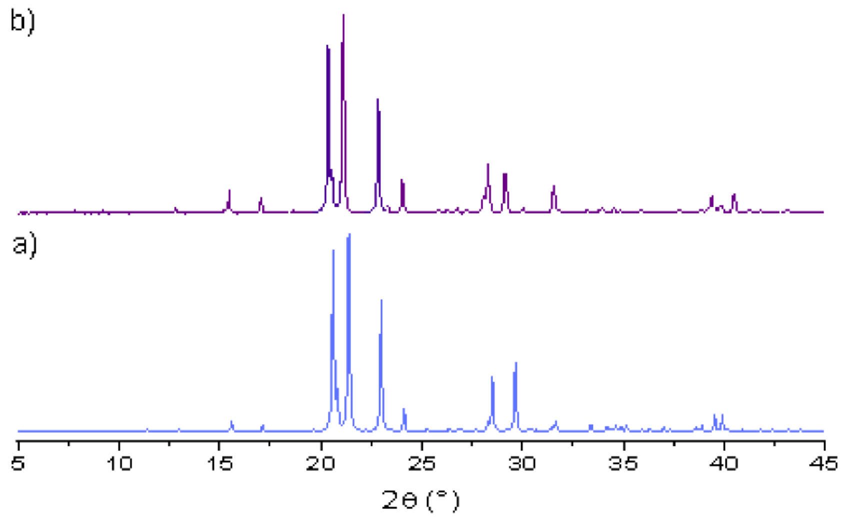

Figure S12. PXRD patterns of compound 5. a) Calculated and b) experimental.

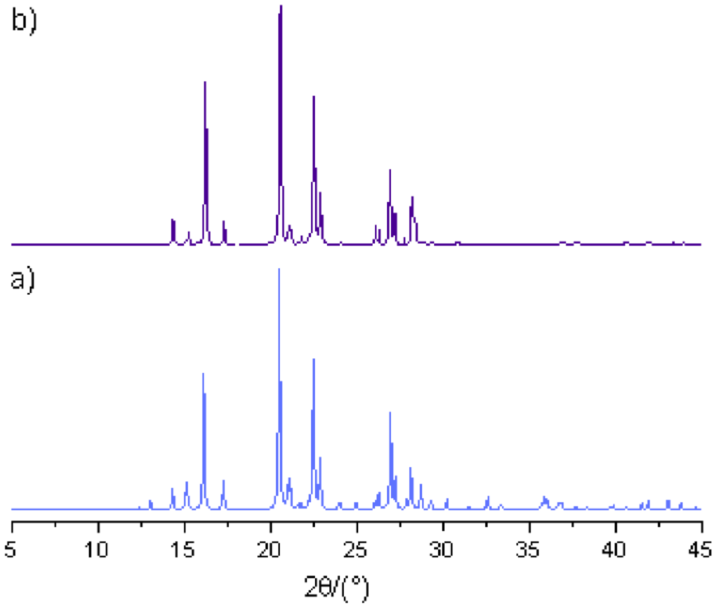

Figure S13. PXRD patterns of compound 6. a) Calculated and b) experimental.

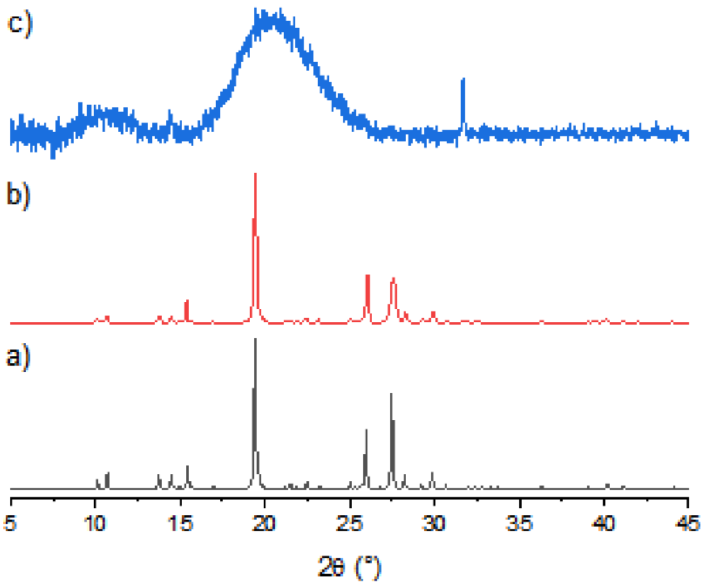

Figure S14. PXRD patterns of compound 3. a) Calculated, b) experimental before UV radiation, and c) experimental after 3h of UV radiation.

S5. NMR spectral data

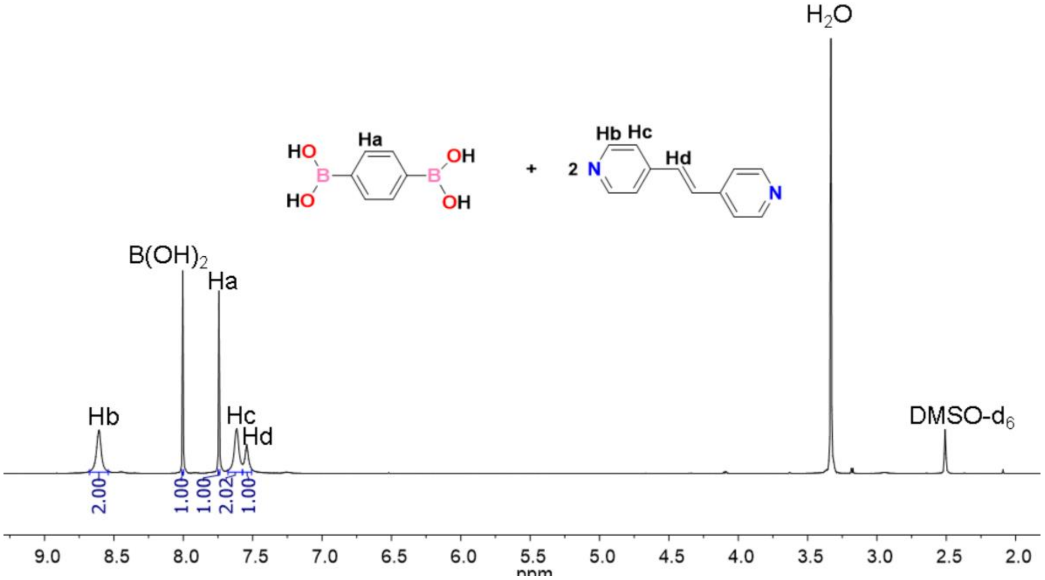

Figure S15.^{1}H NMR spectrum (500 MHz, DMSO-d$_6$) of 1.

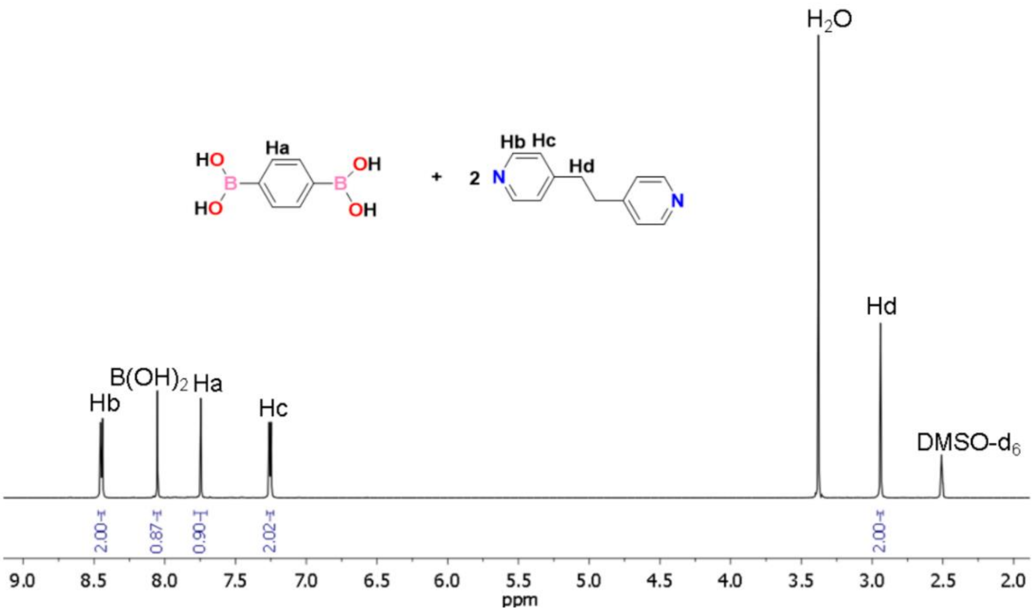

Figure S16. ^{1}H NMR spectrum (500 MHz, DMSO-d_6) of 2.

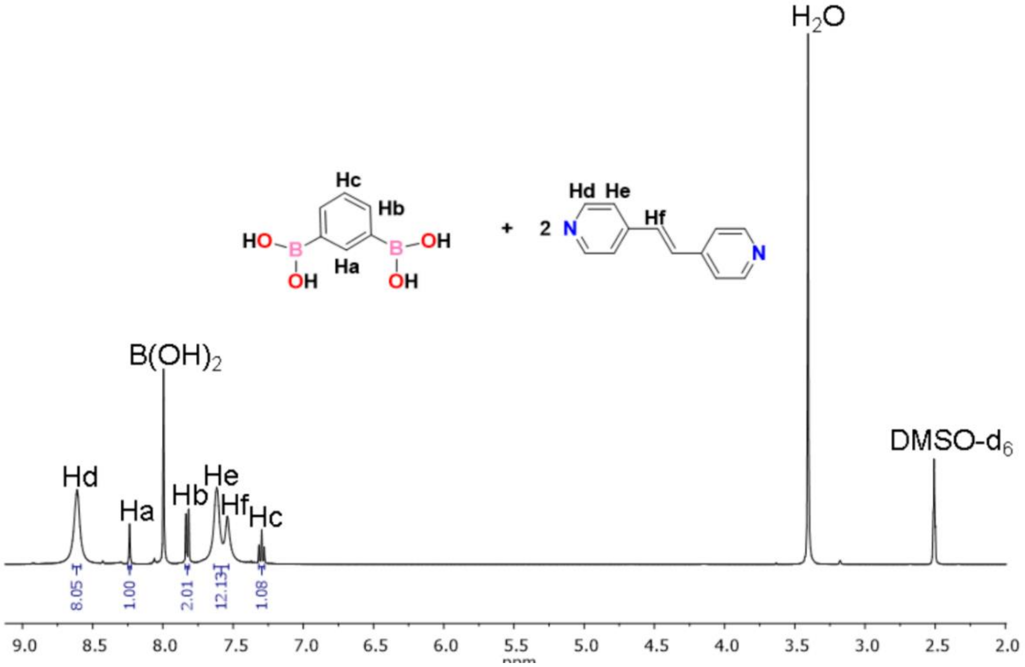

Figure S17. ^{1}H NMR spectrum (500 MHz, DMSO-d_6) of 3.

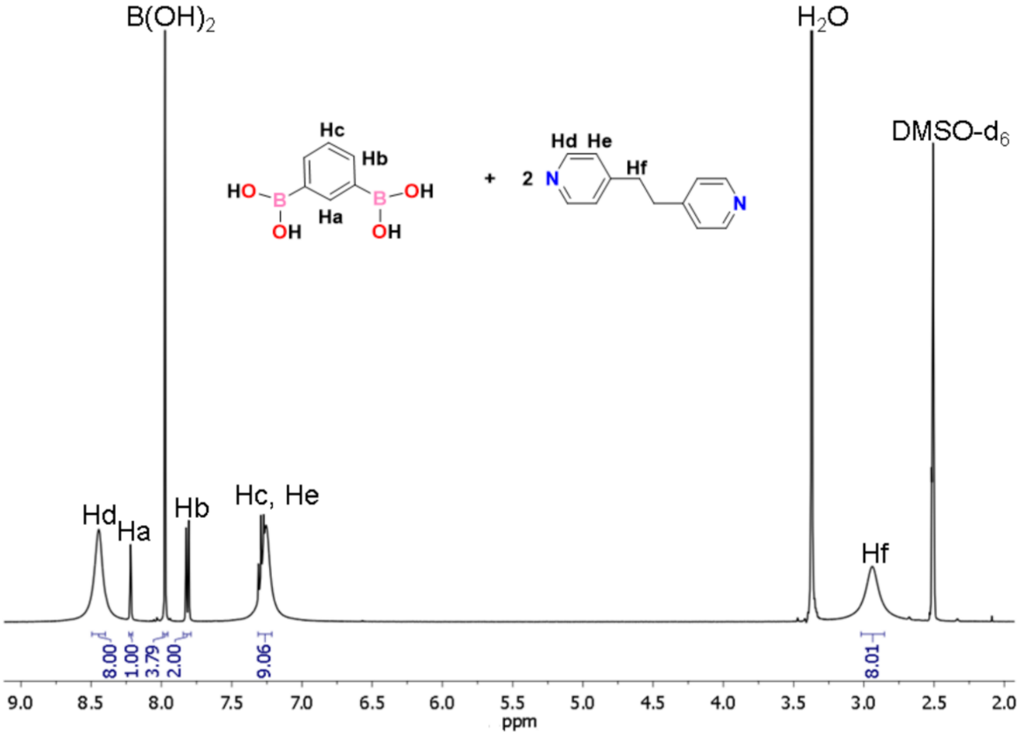

Figure S18. ^{1}H NMR spectrum (500 MHz, DMSO-d_6) of 4.

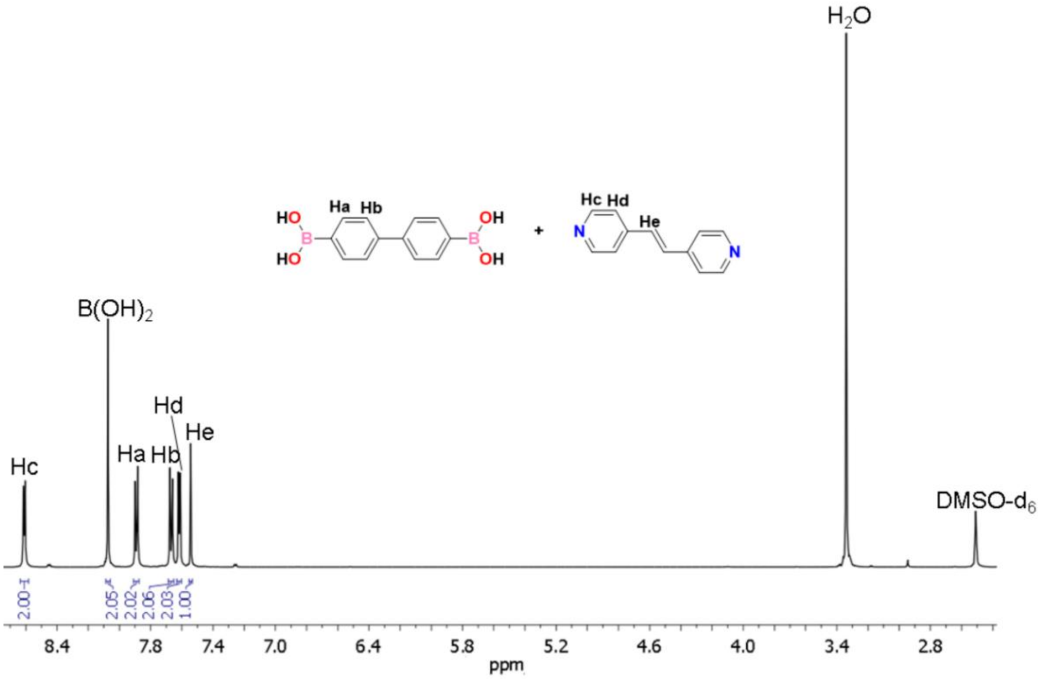

Figure S19. ^{1}H NMR spectrum (500 MHz, DMSO-d_6) of 5.

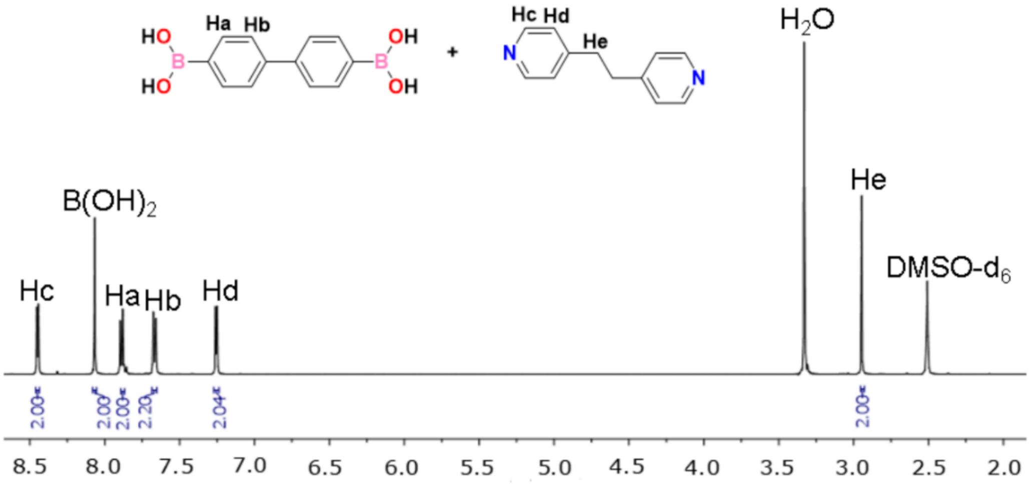

Figure S20. ^{1}H NMR spectrum (500 MHz, DMSO-d$_6$) of 6.

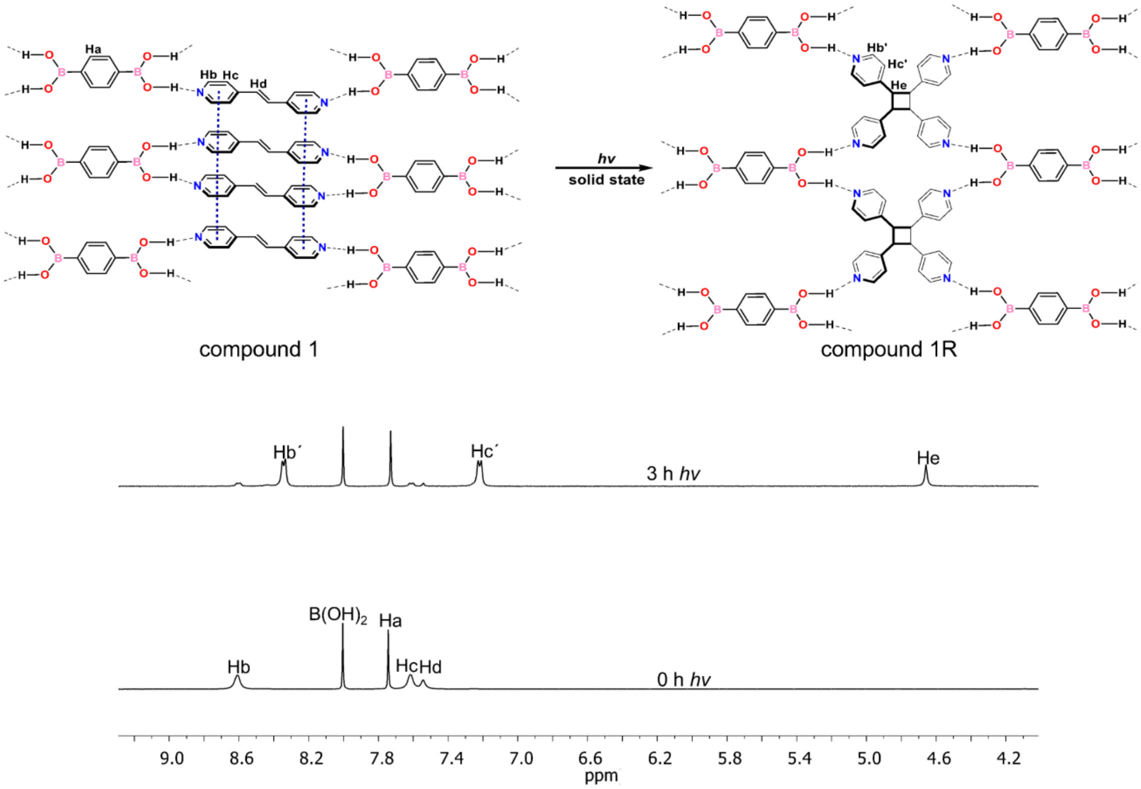

Figure S21. ^{1}H NMR spectra of 1 before (bottom) and after 3 hours UV radiation (top) (500 MHz, DMSO-d$_6$).

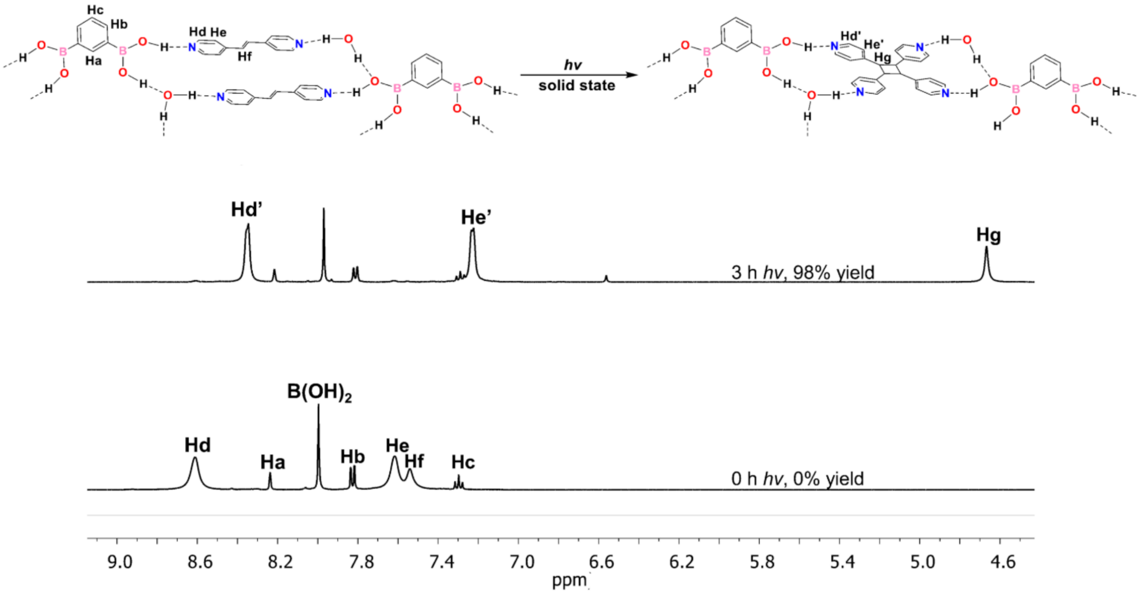

Figure S22. ^{1}H NMR spectra of 3 before (bottom) and after 3 hours UV radiation (top) (500 MHz, DMSO-d_6).

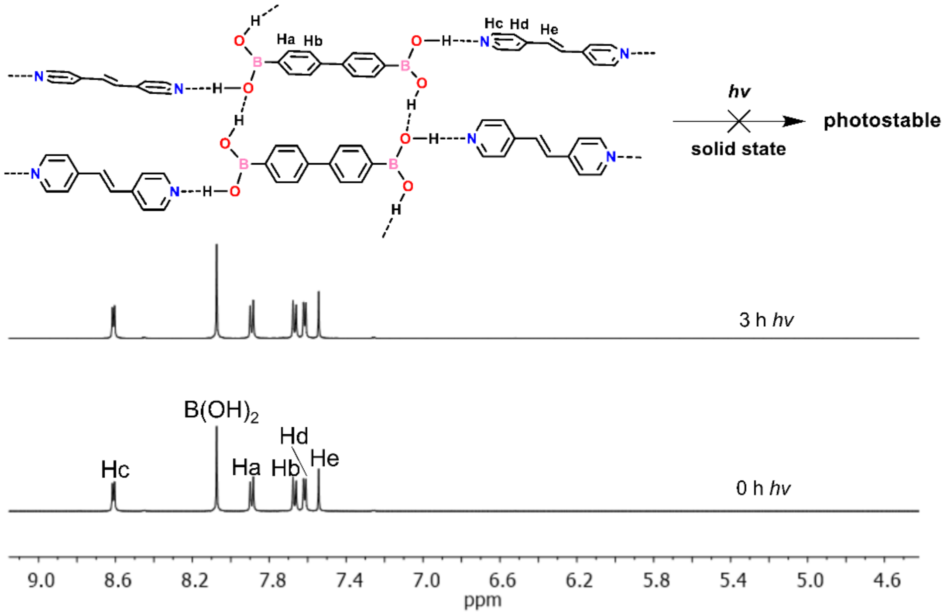

Figure S23. ^{1}H NMR spectra showing photostability of 5 after UV radiation (3 h) (500 MHz, DMSO-d_6).

S6. References

[1] O. V. Dolomanov, L. J. Bourhis, R. J. Gildea, J. A. K. Howard, H. Puschmann, *J. Appl. Cryst.* **2009**, *42*, 339-341.

[2] G. M. Sheldrick, *Acta Cryst*. **2015**, *A71*, 3-8.

[3] G. M. Sheldrick, *Acta Cryst.* **2015**, *C71*, 3-8.

[4] K. Brandenburg, *Diamond*, Crystal Impact GbR, Bonn, Germany, **1997**.
